# CD93 promotes **β**_1_ integrin activation and fibronectin fibrillogenesis during tumor angiogenesis

**DOI:** 10.1172/JCI97459

**Published:** 2018-06-25

**Authors:** Roberta Lugano, Kalyani Vemuri, Di Yu, Michael Bergqvist, Anja Smits, Magnus Essand, Staffan Johansson, Elisabetta Dejana, Anna Dimberg

**Affiliations:** 1 Department of Immunology, Genetics and Pathology, Science for Life Laboratory, Uppsala University, Rudbeck Laboratory, Uppsala, Sweden.; 2 Centre for Research and Development, Uppsala University, Gävle Hospital, Gävle, Sweden.; 3 Department of Radiation Sciences and Oncology, Umeå University Hospital, Umeå, Sweden.; 4 Department of Neuroscience, Neurology, Uppsala University, Uppsala, Sweden.; 5 Institute of Neuroscience and Physiology, Department of Clinical Neuroscience, Sahlgrenska Academy, University of Gothenburg, Gothenburg, Sweden.; 6 Department of Medical Biochemistry and Microbiology, Uppsala University, Uppsala, Sweden.; 7 Vascular Biology Unit, FIRC Institute of Molecular Oncology, Milan, Italy.

**Keywords:** Oncology, Vascular Biology, Brain cancer, Fibronectin, endothelial cells

## Abstract

Tumor angiogenesis occurs through regulation of genes that orchestrate endothelial sprouting and vessel maturation, including deposition of a vessel-associated extracellular matrix. CD93 is a transmembrane receptor that is upregulated in tumor vessels in many cancers, including high-grade glioma. Here, we demonstrate that CD93 regulates β_1_ integrin signaling and organization of fibronectin fibrillogenesis during tumor vascularization. In endothelial cells and mouse retina, CD93 was found to be expressed in endothelial filopodia and to promote filopodia formation. The CD93 localization to endothelial filopodia was stabilized by interaction with multimerin-2 (MMRN2), which inhibited its proteolytic cleavage. The CD93-MMRN2 complex was required for activation of β_1_ integrin, phosphorylation of focal adhesion kinase (FAK), and fibronectin fibrillogenesis in endothelial cells. Consequently, tumor vessels in gliomas implanted orthotopically in CD93-deficient mice showed diminished activation of β_1_ integrin and lacked organization of fibronectin into fibrillar structures. These findings demonstrate a key role of CD93 in vascular maturation and organization of the extracellular matrix in tumors, identifying it as a potential target for therapy.

## Graphical abstract

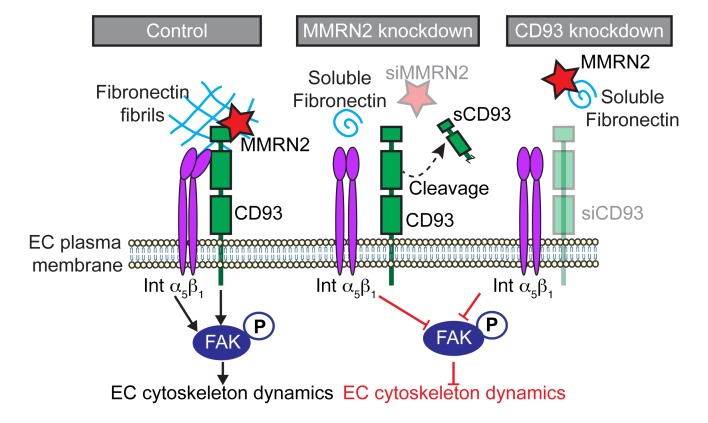

## Introduction

Gliomas are common brain tumors that occur as ependymomas, oligodendrogliomas, and astrocytomas of WHO grades I–IV (1). Glioblastoma (WHO grade IV), the most malignant and common form of glioma, is characterized by microvascular proliferation and morphologically abnormal tumor vessels (2). The aberrant tumor vasculature is permeable and dysfunctional, and contributes to patient morbidity by inducing vasogenic edema in the brain (3). Poor vascular perfusion leads to intermittent hypoxia and a low pH in the tumor microenvironment, and prevents efficient drug delivery, thereby affecting the response to chemotherapy and radiation (4). Therefore, it has been proposed that therapeutic strategies that normalize the tumor vessels may improve the response to conventional cancer therapy (5). Antiangiogenic therapy neutralizing VEGF, or blocking signaling through its cognate receptor VEGFR2, has been approved as second-line treatment for glioblastoma owing to prolonged progression-free survival, although no increase in overall survival is observed (6). Several other angiogenic factors are expressed in glioma and may contribute to tumor angiogenesis and/or resistance to antiangiogenic therapy, including TGF-β2 and pleiotrophin (7, 8). An increased understanding of the mechanisms leading to vascular abnormalities in glioblastoma and its consequences for the tumor microenvironment is necessary to successfully target the tumor vessels. One important step toward this goal is to map proteins specifically expressed in glioblastoma vessels and investigate their role in tumor angiogenesis.

Through laser-capture microdissection of tumor vessels followed by microarray analysis of gene expression, we have previously distinguished a transcriptional signature consisting of 95 genes differentially expressed in human glioblastoma vessels (7). Among these genes, we identified the single-pass transmembrane glycoprotein CD93 as significantly upregulated in glioblastoma vessels compared with vessels in low-grade glioma and in normal brain. CD93 is predominantly expressed in endothelial cells and consists of an extracellular part including a C-type lectin domain, 5 EGF-like repeats, a serine/threonine-rich mucin-like domain, a transmembrane domain, and a short cytoplasmic domain harboring a binding site for moesin. The extracellular domain can be shed and released during inflammation, and soluble CD93 has been proposed to have a proangiogenic effect through its EGF-like repeats (9, 10).

We have previously demonstrated that CD93 is required for tubular morphogenesis, migration, and adhesion of endothelial cells and plays a key role in organizing the endothelial cytoskeleton and cell junctions (11). Consistent with this, an antibody targeting CD93 inhibited formation of tubular structures (12). Importantly, we found that a high expression of CD93 in tumor vessels correlated with shorter survival of patients with astrocytomas of WHO grades III–IV. We also observed that growth of orthotopically implanted syngeneic GL261 glioma was inhibited in CD93^–/–^ mice, and that this was associated with decreased tumor vessel functionality (11). These observations indicate an important role of CD93 during tumor angiogenesis in glioma. CD93 has been shown to interact with moesin via its cytoplasmic domain and with β-dystroglycan in the extracellular matrix (13, 14). However, the mechanisms through which CD93 affects sprouting angiogenesis and the architecture of tumor vessels are still largely unknown.

Here, we demonstrate that CD93 localizes to filopodia during angiogenesis and is specifically expressed in tip cells in endothelial sprouts formed by embryoid bodies. CD93 localization in filopodia is stabilized by interaction with the extracellular matrix–binding protein multimerin-2 (MMRN2), which anchors CD93 to fibronectin and inhibits its proteolytic cleavage. CD93 colocalizes with activated β_1_ integrin chain in endothelial cells, and is required for full β_1_ integrin activation and fibronectin fibrillogenesis in vitro. Consistent with this, the fibronectin network connected to the tumor vasculature is disrupted in intracranially implanted gliomas in CD93^–/–^ mice. Taken together, our data support a key role of CD93 and MMRN2 in fibronectin fibrillogenesis, which is crucial for tumor angiogenesis and vascular integrity. Most importantly, we define a new mechanism of modulation of integrin activity during cancer angiogenesis.

## Results

### CD93 and MMRN2 interact in human endothelial cells and colocalize in tumor vessels.

**Figure 1:**
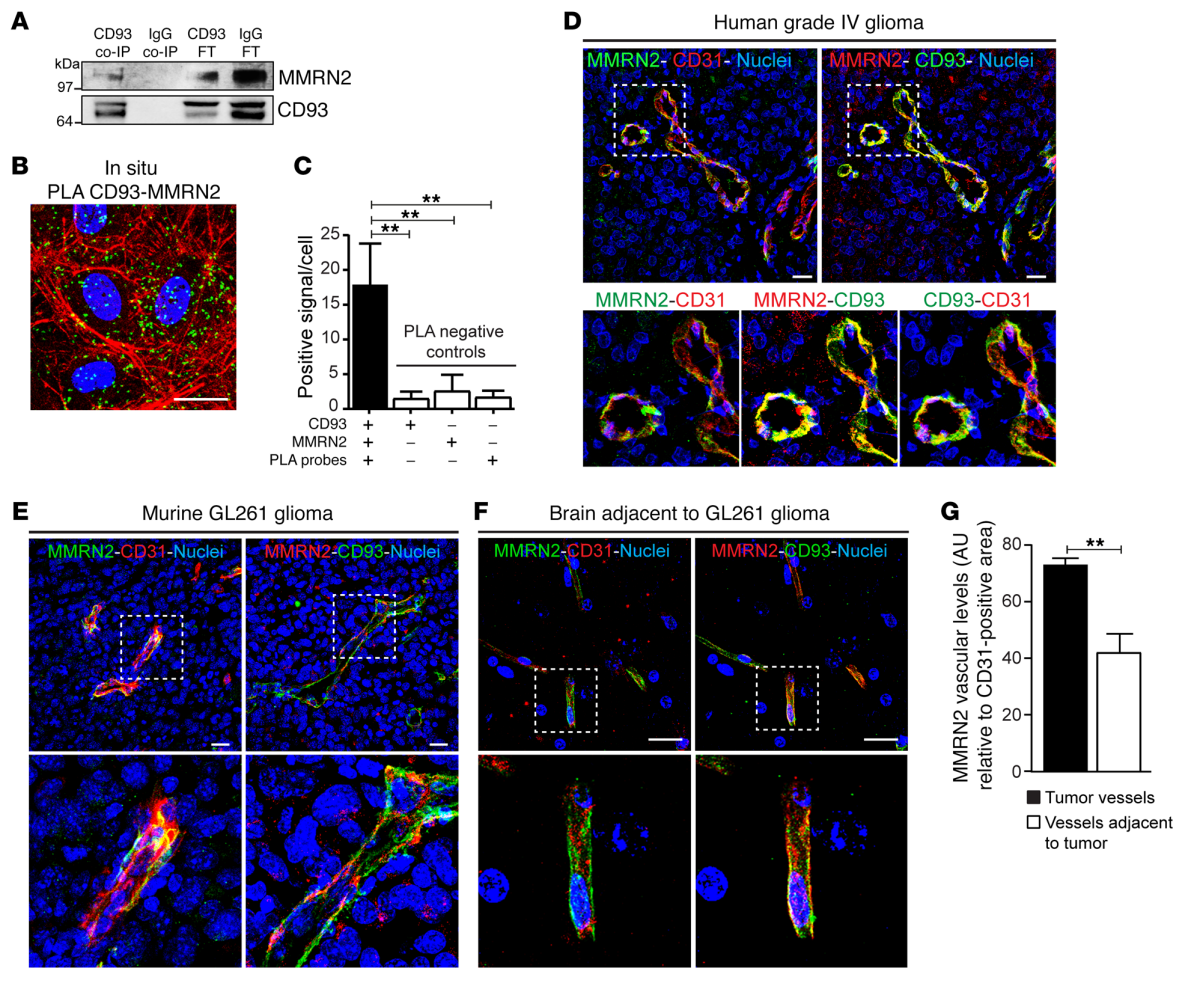
CD93 interacts with MMRN2 in endothelial cells and colocalizes with MMRN2 in tumor vasculature. (**A**) Western blot against MMRN2 in CD93 coimmunoprecipitated samples (CD93 co-IP) derived from total protein lysates of HDMECs. A nonrelevant IgG antibody was used as negative control (IgG co-IP). CD93 FT and IgG FT represent the flow-through of the unbound fraction. (**B**) In situ proximity ligation assay (PLA) for CD93 and MMRN2 in cultured HDBECs. Positive signal (green dots) indicates proximity between CD93 and MMRN2. F-actin was stained by phalloidin (red) and nuclei by Hoechst (blue). Scale bar: 20 μm. (**C**) Quantification of CD93 and MMRN2 interaction based on the number of positive signals per cell (*n* = 3 independent experiments). Nonspecific signals (PLA negative controls) were also examined. **P < 0.01; 1-way ANOVA with Dunnett’s multiple-comparisons test. (**D**–**F**) Immunofluorescent staining of MMRN2, CD93, and CD31 in human grade IV glioma vessels (**D**), in orthotopic GL261 glioma vasculature (**E**), and in nontumor brain vasculature adjacent to a GL261 tumor (**F**). Scale bars in all pictures: 20 μm. (**G**) MMRN2 quantification in tumor and nontumor vessels of WT (*n* = 3) and CD93^–/–^ (*n* = 3) mice. Values represent mean ± SEM expressed as arbitrary units (AU) of MMRN2-positive area normalized by CD31-positive area. **P < 0.01; 2-tailed *t* test.

To investigate the mechanisms through which CD93 regulates vascular function, interacting proteins were identified by coimmunoprecipitation from human endothelial cell protein extracts using a monoclonal anti-CD93 antibody followed by mass spectrometry. Analysis of 5 independent experiments identified the endothelial-specific secreted matrix-binding protein MMRN2 as a potential CD93-binding molecule. MMRN2 was identified in all 5 CD93 coimmunoprecipitated samples and never in samples coimmunoprecipitated with IgG. The CD93-MMRN2 interaction was confirmed by Western blot analysis of CD93 coimmunoprecipitated samples using antibodies directed against MMRN2 (Figure 1A). In situ proximity ligation assay (PLA) technology was used to verify CD93-MMRN2 interaction in human endothelial cell monolayers. Positive PLA signals indicating formation of CD93-MMRN2 complexes were homogeneously distributed in endothelial cells (Figure 1B). The specificity of the PLA assay was confirmed by quantification of CD93-MMRN2 PLA signals per cell, comparing with signals in negative controls (Figure 1C).

CD93 is highly expressed in the tumor vasculature of human high-grade gliomas (15) as well as in tumor vessels in the orthotopic murine GL261 glioma model (11). To determine whether the observed interaction between CD93 and MMRN2 is likely to occur in tumor vessels, we examined the expression pattern of MMRN2 in tumors. MMRN2 was expressed in CD31-positive tumor vessels of human glioblastoma (grade IV glioma), colocalizing with CD93 expression (Figure 1D). Analysis of *y*-*z* 3D stack of the grade IV glioma vessels revealed that MMRN2 and CD93 were expressed at the abluminal side of the CD31-positive glioblastoma vessels (arrowheads in Supplemental Figure 1, A and B, respectively; supplemental material available online with this article; https://doi.org/10.1172/JCI97459DS1), indicating that the interaction between MMRN2 and CD93 occurs abluminally in proximity to extracellular matrix (arrowheads in Supplemental Figure 1, C and D). Similarly, MMRN2 was highly expressed in murine GL261 glioma vasculature, colocalizing with CD93 (Figure 1E). A significantly lower basal expression of MMRN2 colocalizing with CD93 was observed in the brain vasculature adjacent to the GL261 tumor (Figure 1F and quantification in Figure 1G).

### CD93 colocalizes with MMRN2 during retinal angiogenesis and regulates filopodia formation and vessel sprouting.

**Figure 2:**
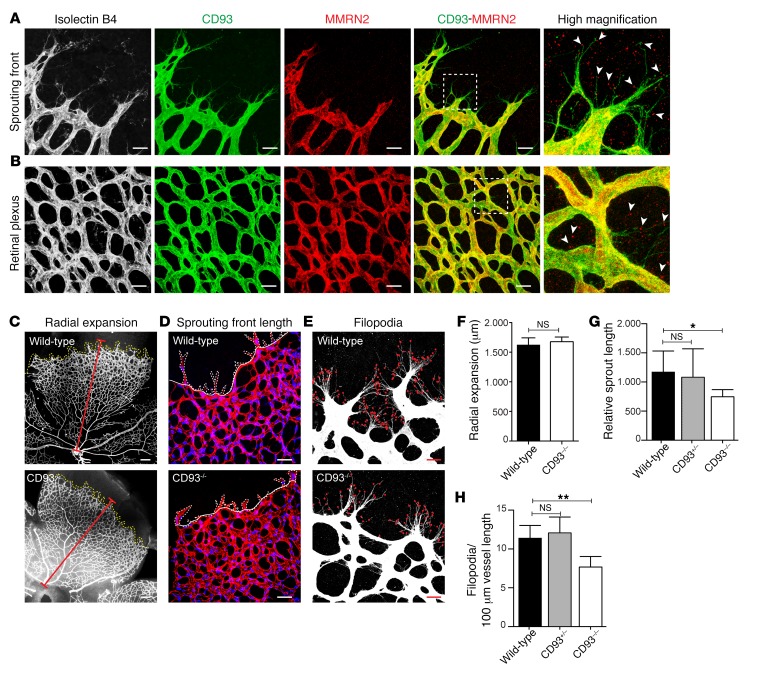
CD93 colocalizes with MMRN2 in growing retinal vasculature and regulates filopodia protrusions and vessel sprouting. (**A**) CD93 and MMRN2 immunofluorescent staining in the sprouting front of P6 WT mouse retina. The vasculature is visualized by isolectin B4. Scale bars: 20 μm. High-magnification picture shows CD93 localized in the filopodia and CD93/MMRN2 colocalization in the sprouting front. Arrowheads indicate secreted MMRN2 residing in the extracellular matrix close to the filopodia. (**B**) CD93 and MMRN2 staining in the retinal plexus region. High magnification shows CD93/MMRN2 colocalization in the vasculature. Arrowheads indicate secreted MMRN2. Scale bars: 20 μm. (**C**) Radial expansion of vessels in WT and CD93^–/–^ mouse retina. The red line indicates the extension of the vascular network. Scale bars: 150 μm. (**D**) Vascular sprouts at the front of the retinal vasculature in WT and CD93^–/–^ mice. The length of the endothelial sprouting front is indicated by a dotted line. Nuclei were visualized by ERG (blue). Scale bars: 50 μm. (**E**) Filopodia protrusions in WT and CD93^–/–^ mice. Red dots indicate filopodia in the sprouting front. Scale bars: 20 μm. (**F**) Quantification of radial expansion in WT (*n* = 14) and CD93^–/–^ (*n* = 7) mice. Values indicate the vessel migration length expressed in micrometers. Values represent mean ± SEM; 2-tailed *t* test. (**G**) Quantification of the sprouting front length in WT (*n* = 7), CD93^+/–^ (*n* = 4), and CD93^–/–^ (*n* = 7) mice. Values indicate the length of the sprouts relative to the migrating front length. (**H**) Filopodia quantification in WT (*n* = 5), CD93^+/–^ (*n* = 4), and CD93^–/–^ (*n* = 5) mice. Values indicate the number of filopodia per 100 μm vessel length. *P < 0.05, **P < 0.01; 1-way ANOVA with Dunnett’s multiple-comparisons test.

To further understand the role of CD93 in sprouting angiogenesis, we analyzed the developing vasculature in mouse retina. CD93 was expressed in the sprouting front of the postnatal day 6 (P6) mouse retinas, including the filopodia protrusions, colocalizing with the endothelial marker isolectin B4 (Figure 2A). MMRN2 colocalized with CD93 in the retinal plexus and sprouting front, but colocalization was not detectable in filopodia extensions (high magnification, Figure 2, A and B). Instead, MMRN2-positive signals were found in the extracellular matrix surrounding the filopodia at the vascular front (arrowheads, Figure 2A) and within the vascular plexus (arrowheads, Figure 2B). This indicates that MMRN2 is not present within the filopodia, but interacts with CD93 in filopodia after being secreted. To investigate whether loss of CD93 affects retinal angiogenesis, we analyzed P6 retinas from CD93-deficient (CD93^–/–^) mice and WT littermates. The radial expansion of the vascular plexus was similar in CD93^–/–^ and WT retinas (Figure 2C, quantified in Figure 2F). However, the mean length of the sprouts in the angiogenic front was significantly reduced in the CD93^–/–^ retinal vasculature in comparison with WT littermates (Figure 2D, quantified in Figure 2G). In addition, a significant reduction in filopodia protrusions was observed in CD93^–/–^ mice compared with WT mice (Figure 2E, quantified in Figure 2H). No differences were observed when the sprout length and the number of filopodia were compared between WT and CD93 heterozygous mice (CD93^+/–^; Figure 2, G and H). The importance of CD93 in filopodia formation was further analyzed through siRNA-mediated knockdown of CD93 in human dermal blood endothelial cells (HDBECs). In line with a reduced number of filopodia in CD93^–/–^ P6 retinas, sparsely seeded CD93 siRNA-treated endothelial cells formed fewer filopodia than control cells (Supplemental Figure 2, A and B). These data indicate that CD93 regulates filopodia formation and the extension of endothelial sprouts during angiogenesis.

### MMRN2 protects CD93 from proteolytic cleavage.

**Figure 3:**
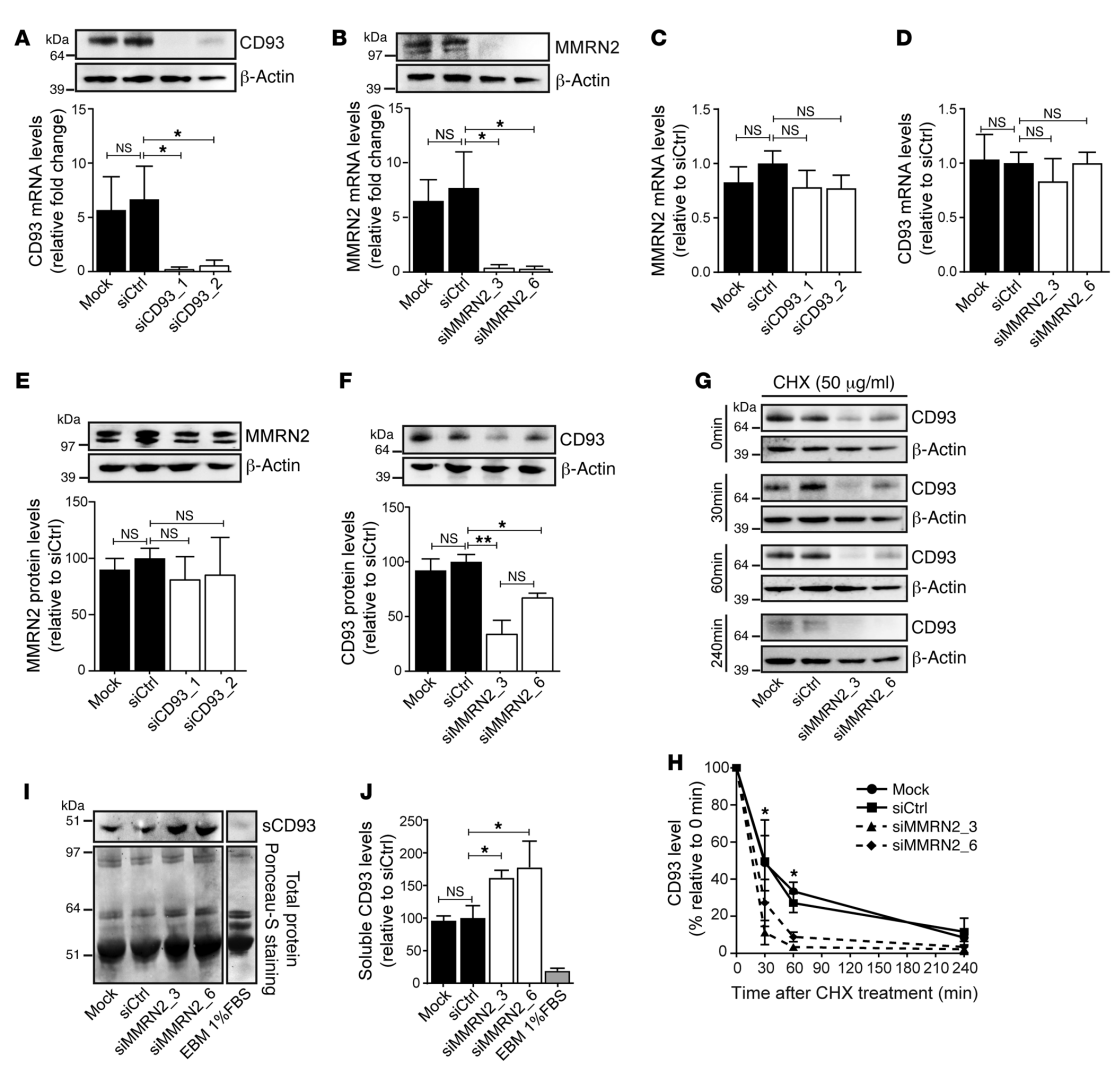
MMRN2 downregulation impairs CD93 protein stability in endothelial cells. (**A**) Western blot and real-time quantitative PCR (qPCR) showing the efficiency of CD93 knockdown in HDBECs (siCD93_1 and siCD93_2) at 48 hours after siRNA transfection. Untransfected cells (Mock) and scramble siRNA-transfected cells (siCtrl) were used as controls. (**B**) Western blot and real-time qPCR for MMRN2 showing the efficiency of MMRN2 knockdown in HDBECs (siMMRN2_3 and siMMRN2_6). (**C**) MMRN2 mRNA levels after CD93 knockdown in HDBECs. (**D**) CD93 mRNA level after MMRN2 knockdown. Values represent fold change relative to siCtrl-transfected cells. (**E**) Western blot against MMRN2 in siCD93-transfected cells and controls. β-Actin was used as loading control. (**F**) Western blot against CD93 and β-actin in siMMRN2-transfected cells and controls. (**G**) Western blot against CD93 in control and siMMRN2-silenced HDBECs in the presence or absence of 50 μg/ml cycloheximide (CHX). The stability of CD93 in control cells and in siMMRN2-silenced cells was assessed at different time points after CHX treatment (0, 30, 60, and 240 minutes). (**H**) Quantification of CD93 levels during specific time points in the presence of 50 μg/ml CHX in control cells and siMMRN2-silenced cells. Values represent the percentage of CD93 relative to CHX-untreated cells in all conditions (control and siMMRN2 cells). *P < 0.05; 2-way ANOVA with Tukey’s post-test. Values represent mean ± SEM (3 independent experiments). (**I**) Representative Western blot of soluble CD93 (sCD93) levels in conditioned media supplemented with 1%FBS (EBM 1%FBS) derived from control and siMMRN2-silenced cells. (**J**) Quantification of sCD93 levels in the conditioned media derived from control and siMMRN2-treated cells. Values shown in the quantification graphs in **A**–**F** and **J** represent mean ± SEM (3 independent experiments); *P < 0.05, **P < 0.001; 1-way ANOVA with Dunnett’s multiple-comparisons test.

To investigate whether the interaction between CD93 and MMRN2 affects the expression or stability of either protein, CD93 and MMRN2 mRNA and protein levels were examined in HDBECs after siRNA-mediated knockdown of CD93 or MMRN2. Transfection of endothelial cells with siRNA targeting CD93 reduced CD93 mRNA expression and protein level by approximately 95% compared with controls (Figure 3A). An equally high knockdown efficiency was obtained using siRNAs against MMRN2 (Figure 3B). siRNA-mediated knockdown of CD93 did not affect MMRN2 mRNA or protein levels (Figure 3, C and E). Similarly, CD93 mRNA levels were not affected by MMRN2 downregulation (Figure 3D). However, Western blot analysis revealed a significant reduction of CD93 protein levels in siMMRN2-treated cells compared with controls (Figure 3F). This suggests that MMRN2 affects CD93 protein stability but not CD93 gene expression.

To determine whether MMRN2 regulates CD93 protein stability in endothelial cells, protein synthesis was blocked with cycloheximide (CHX) treatment over a time course of 0, 30, 60, and 240 minutes in siMMRN2 and control cells, and CD93 protein levels were determined by Western blot analysis. A gradual reduction of CD93 over time was observed in the control conditions (Mock and siCtrl) in response to CHX treatment, indicating a time-dependent turnover of CD93 protein (Figure 3G and graph in Figure 3H). When MMRN2 expression was knocked down by siRNA treatment, the reduction of CD93 protein levels was accelerated (Figure 3, G and H). These results indicate that the interaction with MMRN2 stabilizes CD93 protein in endothelial cells.

To further characterize the mechanism by which MMRN2 regulates CD93 protein levels, we assessed the possible impact of MMRN2 in CD93 degradation, using proteasome and lysosome inhibitors. Inhibition of either proteasomal degradation by 20 μM lactacystin during 8 hours or lysosomal degradation using 10 mM NH_4_Cl during 18 hours resulted in accumulation of CD93 protein in controls, indicating that CD93 is degraded through both proteasome- and lysosome-dependent mechanisms (Supplemental Figure 3, A and B). However, the siMMRN2-mediated decrease in CD93 protein level was not restored to control levels by treatment with either proteasome or lysosome inhibitors. These results indicate that the reduction of CD93 protein levels observed after MMRN2 knockdown is not due to enhanced intracellular protein degradation.

CD93 can be cleaved from the plasma membrane in response to inflammatory conditions, releasing a soluble CD93 fragment containing the EGF-like domain (9). We therefore assessed whether the interaction with MMRN2 affected shedding of CD93 from endothelial cells. Conditioned media from control cells and siMMRN2-treated cells were concentrated and analyzed by Western blot to detect soluble CD93 (Figure 3I). Notably, the level of soluble CD93 was increased more than 60% in the conditioned medium derived from siMMRN2-treated cells as compared with the soluble CD93 levels in that derived from control cells (Figure 3J). These results indicate that MMRN2 protects CD93 from cleavage in endothelial cells, and that decreased expression of MMRN2 leads to an increased release of soluble CD93.

### MMRN2 stabilizes CD93 in filopodia and in the front edge of migrating endothelial cells.

**Figure 4:**
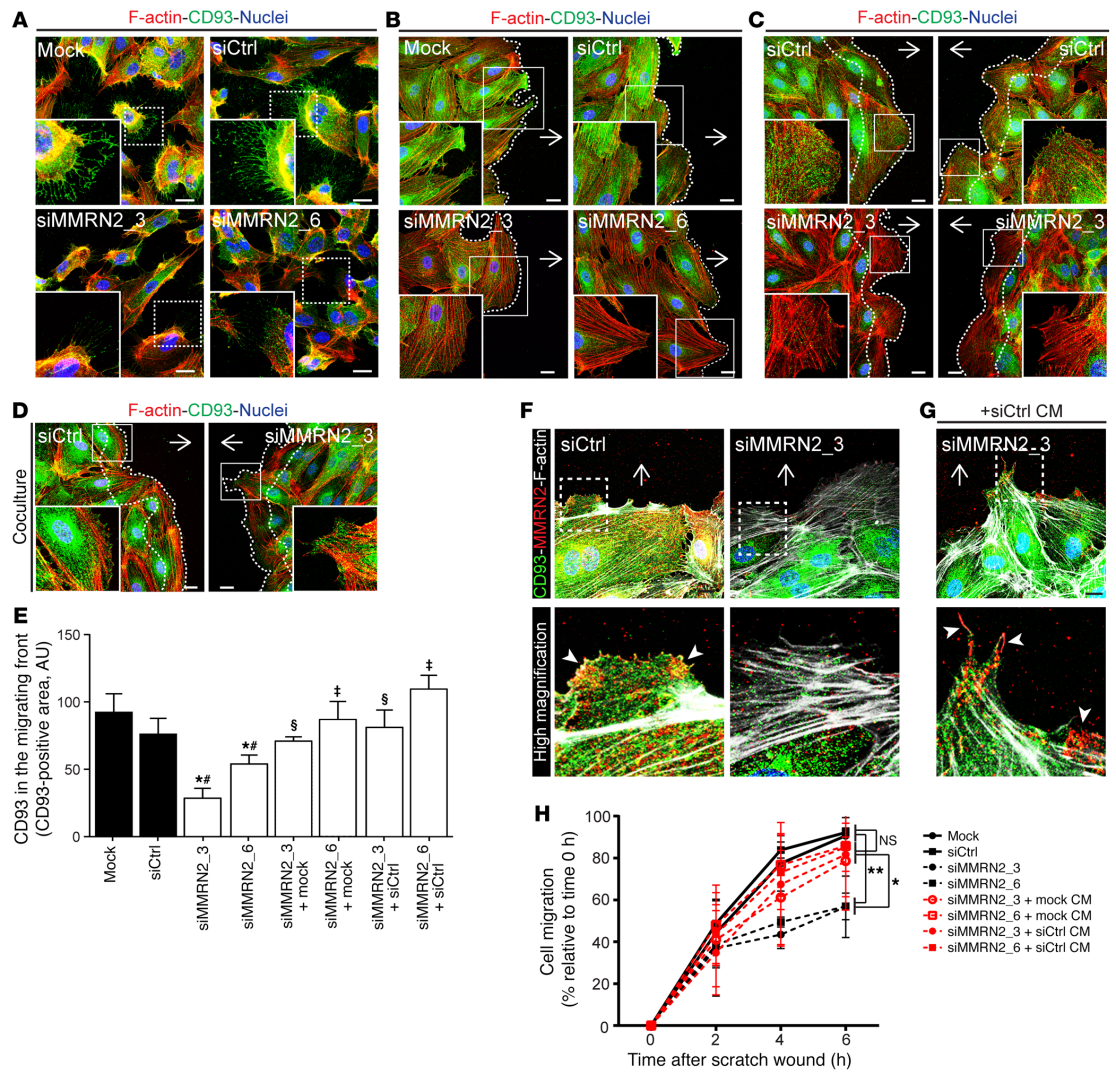
MMRN2 stabilizes CD93 in the filopodia and in the migrating front of HDBECs. (**A**) Immunofluorescent staining of CD93 (green), phalloidin (red), and nuclei (blue) in control (Mock and siCtrl) and MMRN2-silenced (siMMRN2_3 and siMMRN2_6) HDBECs cultured under nonconfluent conditions. High-magnification images show localization of CD93 in the filopodia. Scale bars: 20 μm. (**B**) CD93 (green) in the migrating front of control and siMMRN2-silenced cells at 6 hours after production of a double-sided scratch in the cell monolayer. Arrows indicate direction of migration. High-magnification pictures show CD93 localization in the migrating front of control and siMMRN2 cells. Scale bars: 20 μm. (**C** and **D**) Rescue experiment performed using a 2-well culture insert enabling coculturing of control cells and siMMRN2-transfected cells during migration. Immunofluorescent staining of CD93 (green), phalloidin (red), and nuclei (blue). Scale bars: 20 μm. CD93 localization was assessed after 6 hours of migration in control and siMMRN2 cells (**C**) and siMMRN2 cells cocultured with control cells (**D**). (**E**) Quantification of CD93 in the migrating front area. Bars represent arbitrary units (AU) of the CD93-positive signal at the migrating front area depicted by dotted line in **C** and **D**. *P < 0.05 vs. Mock, ^#^*P* < 0.05 vs. siCtrl, ^§^vs. siMMRN2_3, ^‡^vs. siMMRN2_6; 1-way ANOVA with Dunnett’s post-test. Values represent mean ± SEM (4 different areas along the migrating edge in 3 independent experiments). (**F** and **G**) Costaining of CD93 (green), MMRN2 (red), and phalloidin (gray) in the migrating front of control and siMMRN2 cells and siMMRN2 cells cultured during 24 hours in conditioned medium derived from control cells (+siCtrl CM). Scale bars: 20 μm. (**H**) Migration assay in control cells, MMRN2-downregulated cells, and siMMRN2-transfected cells cultured in control conditioned medium (CM). Values represent mean ± SEM (3 independent experiments); *P < 0.05, **P < 0.01; 1-way ANOVA with Dunnett’s multiple-comparisons test.

We have previously demonstrated that CD93 is required for endothelial cell adhesion and migration (11). To investigate whether the interaction with MMRN2 decreases cleavage of CD93 in endothelial filopodia, the subcellular localization of CD93 was assessed by confocal microscopy in sparsely cultured HDBECs transfected with siRNA targeting MMRN2 or control siRNA. In line with our observation of CD93 expression in filopodia in endothelial sprouts of the developing retinal vasculature (Figure 2), CD93 was localized in filopodia structures in control cells (Figure 4A). Downregulation of MMRN2 resulted in a striking decrease of CD93 in filopodia. In siMMRN2-transfected cells, CD93 was mainly accumulated in the perinuclear region, and only low amounts were detected in filopodia (Figure 4A). The only partial reduction of CD93 in filopodia did not result in a decreased number of filopodia in siMMRN2-transfected HDBECs, which is consistent with similar numbers of filopodia in the retinas of CD93^+/–^ and WT mice (Supplemental Figure 4, A and B, and Figure 2, G and H).

To further analyze the localization of CD93 during endothelial cell migration, an in vitro wound healing assay was used, and the localization of CD93 in the leading edge of migrating cells was analyzed by confocal microscopy. A strong CD93 staining was consistently detected in the migrating front and filopodia of control endothelial cells (Figure 4B). In contrast, only low levels of CD93 were observed at the migration front in MMRN2-silenced cells. The CD93-positive signal in siMMRN2-treated endothelial cells was mainly located in the perinuclear region (Figure 4B).

MMRN2 is an extracellular matrix protein secreted by endothelial cells. Therefore, we analyzed whether MMRN2 produced and secreted by control cells can rescue CD93 localization to endothelial filopodia and the migrating front in siMMRN2-transfected cells. The rescue experiment was performed using cell culture inserts. Control cells and siMMRN2 cells were initially cultured in separate wells until confluence. When the culture insert was removed, control and siMMRN2-treated endothelial cells were allowed to migrate for 6 hours, sharing the same culture media (Supplemental Figure 4C). CD93 was strongly localized in the migrating front and filopodia when control endothelial cells were cocultured on each side of the insert. However, a significant reduction of CD93-positive signal was detected in the migrating front when siMMRN2-treated cells were present on both sides of the insert (Figure 4C). Coculture with control cells restored CD93 levels in the migrating front of MMRN2-silenced cells (Figure 4D). CD93-positive signals in the migrating front (area delimited by dotted line in Figure 4, C and D) were quantified, showing a complete rescue of CD93 levels in MMRN2 siRNA-treated endothelial cells when cocultured with control endothelial cells (Figure 4E). Thus, MMRN2 secreted by control cells can stabilize CD93 in the migrating front and filopodia of MMRN2-deficient endothelial cells. This indicates that CD93 in filopodia interacts with MMRN2 in the extracellular matrix. This result was further confirmed by costaining of CD93 and MMRN2 during migration. MMRN2 colocalized with CD93 in the migrating front in the control condition (arrowheads in the high-magnification picture of siCtrl, Figure 4F). Knockdown of MMRN2 resulted in a loss of CD93 in the migrating front (siMMRN2_3 panel, Figure 4F). CD93 localization to filopodia is rescued by treatment of siMMRN2-treated endothelial cells with conditioned medium (CM) derived from control cells. In association, we observed that MMRN2, derived from conditioned medium, bound to endothelial filopodia on siMMRN2-treated cells (arrowheads in siMMRN2_3+siCtrl CM, Figure 4G). As a functional readout, we quantified endothelial migration using the scratch wound assay. Downregulation of MMRN2 (siMMRN2_3 and siMMRN2_6, black dashed lines, Figure 4H) significantly impaired migration at 6 hours after wounding in comparison with control cells (Mock and siCtrl, solid black lines, Figure 4H). This inhibitory effect was reverted by culturing of siMMRN2 cells in conditioned media from control cells containing secreted MMRN2 (siMMRN2+Mock or siCtrl CM, red dashed lines, Figure 4H).

### CD93 is predominantly expressed in tip cells, while MMRN2 is distributed throughout endothelial sprouts in embryoid bodies.

**Figure 5:**
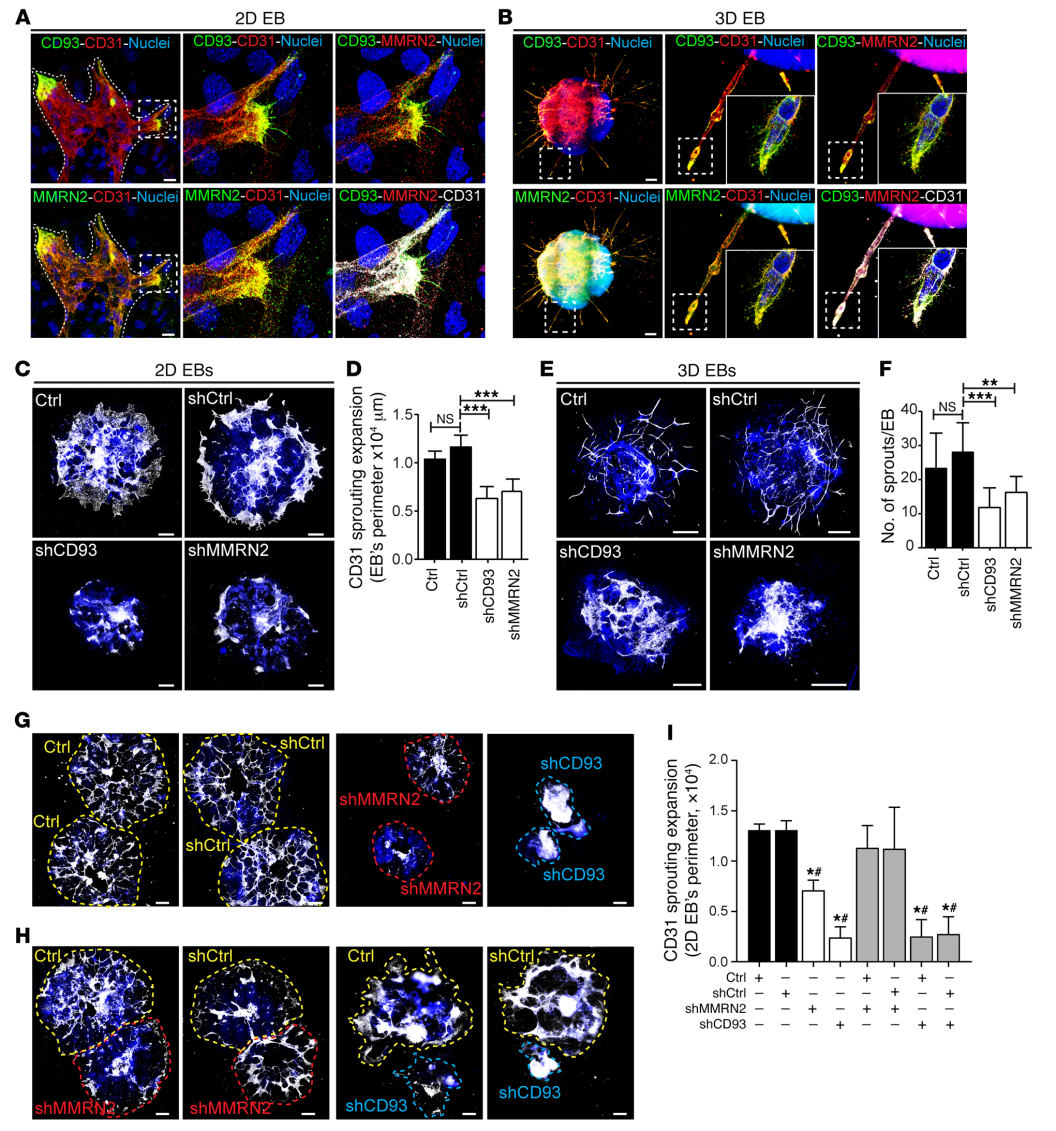
CD93 and MMRN2 colocalize in the tip cells during sprouting angiogenesis and are required for endothelial sprouting. (**A**) Immunofluorescent staining of CD93 and MMRN2 in the sprouting front (indicated by dotted line) of vessels formed in embryoid bodies (EBs) cultured in 2D on glass slides upon VEGF treatment. Differentiated endothelial cells were visualized by CD31 staining. Scale bars: 20 μm. Specific localization of CD93 and MMRN2 in CD31-positive sprouts and filopodia is shown in high-magnification images. (**B**) Immunofluorescent staining of CD93 and MMRN2 in EBs cultured in a 3D collagen gel. Scale bars: 200 μm. High-magnification images show a specific localization of CD93 in the tip cells and in filopodia. MMRN2 staining indicates its colocalization with CD31-positive sprouts and with CD93 in the tip cells. (**C**) CD31 immunofluorescent staining of shCD93- and shMMRN2-transfected EBs cultured in 2D glass slide. Untransfected (Ctrl) or empty vector–transfected (shCtrl) EBs were used as controls. Scale bars: 500 μm. (**D**) Quantification of CD31 sprouting expansion. Values represent mean ± SEM (at least *n* = 5 EBs per condition). ***P < 0.001; 1-way ANOVA with Dunnett’s multiple-comparisons test. (**E**) CD31 immunofluorescent staining of shCD93- and shMMRN2-transfected or control EBs (Ctrl and shCtrl) cultured in 3D collagen gel. Scale bars: 500 μm. (**F**) Quantification of CD31-positive sprouts. Values represent mean ± SEM (at least *n* = 10 EBs per condition). **P < 0.01, ***P < 0.001; 1-way ANOVA with Dunnett’s multiple-comparisons test. (**G**) Coculture of 2 individual EBs of each condition in 2D glass slide. (**H**) Coculture of control EBs (Ctrl or shCtrl) with shMMRN2 or shCD93 EBs. (**I**) Quantification of the CD31 sprouting expansion rescued upon coculture of control EBs with shMMRN2 or shCD93 EBs. *P < 0.05 vs. Ctrl; ^#^*P* < 0.05 vs. shCtrl; 1-way ANOVA with Dunnett’s multiple-comparisons test.

We used the embryoid body (EB) model of angiogenesis to examine the expression patterns of CD93 and MMRN2 during blood vessel formation. EBs are aggregates of embryonic stem (ES) cells that can be induced to differentiate into various cell types and, when cultured in the presence of VEGF, recapitulate sprouting angiogenesis (16). EBs were grown on a 2-dimensional (2D) glass slide surface or in a 3D collagen I matrix and stimulated with VEGFA to induce sprouting angiogenesis. Immunofluorescent staining of EBs cultured on 2D glass slide surfaces showed that CD93 was exclusively localized in the sprouting front of the vascular plexus with high levels detected in endothelial filopodia (Figure 5A). In contrast, MMRN2 expression was observed throughout the vascular network and colocalized with CD93 in the sprouting front (high magnification in Figure 5A). MMRN2 was specifically expressed in endothelial cells within the retina, consistent with previous publications (17, 18). A similar localization of CD93 and MMRN2 was observed in sprouting vessels formed by EBs cultured in a 3D collagen I matrix (Figure 5B). CD93 was mainly observed in the tip cells of endothelial sprouts, whereas MMRN2 was expressed both in the tip and in the stalk cells, colocalizing with CD93 in the tip cell filopodia (Figure 5B).

### CD93 and MMRN2 are required for endothelial sprouting in EBs.

To determine the necessity of CD93 and MMRN2 for sprouting angiogenesis, ES cells were transduced with lentiviruses expressing shRNAs designed to knock down CD93 (shCD93) or MMRN2 (shMMRN2). Empty vector–transduced (shCtrl) and untransduced cells (Ctrl) were used as controls. Transfection of ES cells with shRNA targeting CD93 or MMRN2 reduced the mRNA expression of CD93 and MMRN2 by approximately 90% compared with controls in EBs (Supplemental Figure 5, A and B). GFP detection indicating shRNA-transduced cells showed an efficient transduction in 2D and 3D EBs (Supplemental Figure 5, C and D). Knockdown of either CD93 or MMRN2 in ES cells was associated with deficient formation of endothelial sprouts in VEGF-treated EBs (Figure 5, C–F). Indeed, the area of CD31-positive sprouts was significantly decreased in 2D EBs when either CD93 or MMRN2 was knocked down (Figure 5, C and D). Similarly, shCD93 and shMMRN2 EBs cultured in 3D collagen matrix showed a significant inhibition in the number of endothelial sprouts as compared with controls (Figure 5, E and F).

Consistent with a role of secreted MMRN2 in interacting with and stabilizing CD93 during endothelial migration and sprouting, deficient sprouting in 2D shMMRN2 EBs (Figure 5G) could be rescued by coculturing with control EBs (Figure 5, G and H, quantified in Figure 5I). In contrast, endothelial sprouting in CD93-deficient EBs was not rescued by coculturing with control EBs, consistent with a key role of cell surface–bound CD93 in this context (Figure 5, G and H, quantified in Figure 5I). These results support an important role of CD93 and MMRN2 during sprouting angiogenesis.

### CD93 or MMRN2 downregulation disrupts fibronectin fibrillogenesis in endothelial cells.

**Figure 6:**
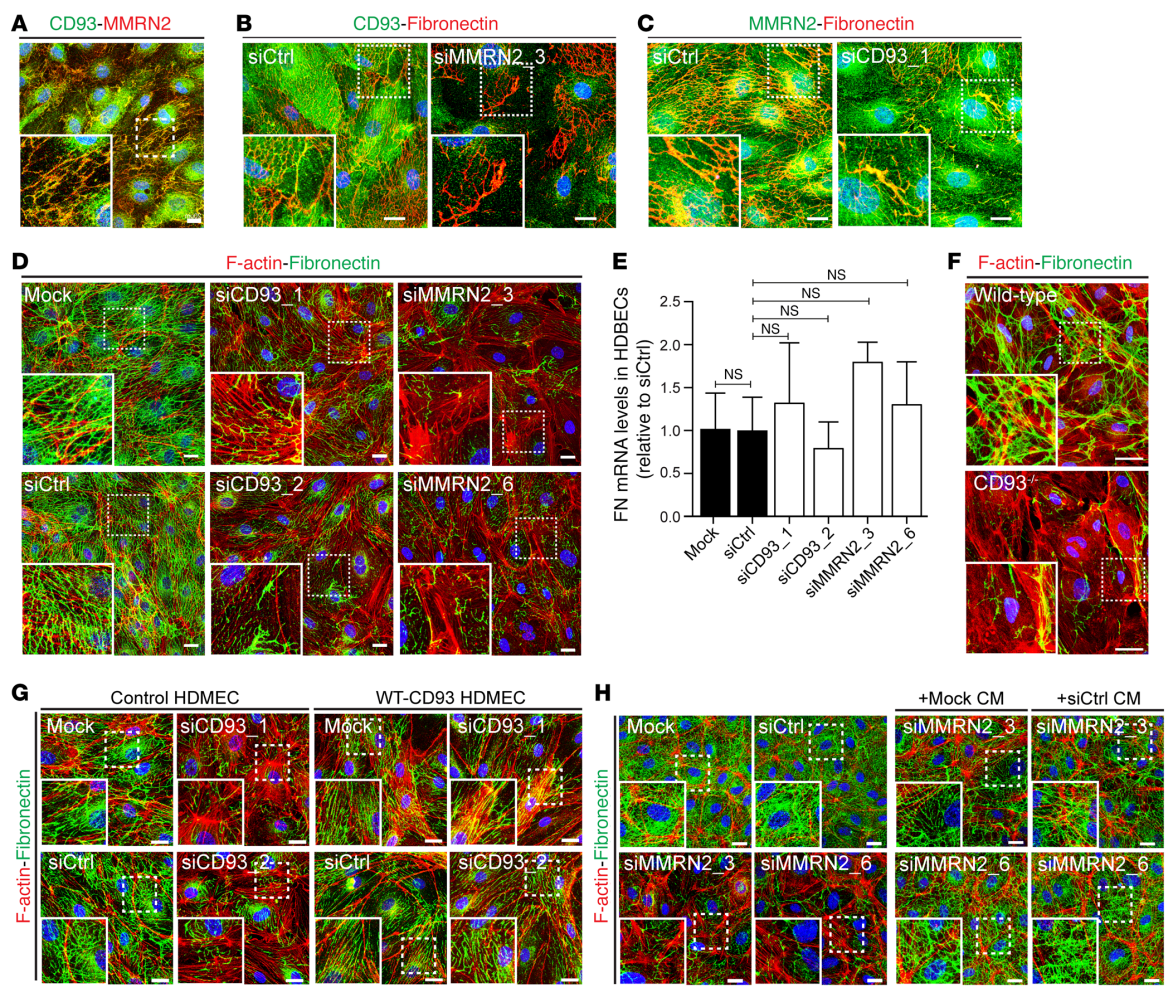
CD93 and MMRN2 regulate fibronectin fibrillogenesis in endothelial cells. (**A**) CD93 (green) and MMRN2 (red) colocalization in confluent HDBECs. High-magnification picture shows CD93-MMRN2 colocalizing in fibrillary structures. Scale bar: 20 μm. (**B**) CD93 (green) and fibronectin (red) costaining in control or MMRN2-silenced (siMMRN2_3) confluent HDBECs. Scale bars: 20 μm. (**C**) MMRN2 (green) and fibronectin (red) costaining in control or CD93-downregulated cells (siCD93_1). (**D**) Immunofluorescent staining of fibronectin (green) and F-actin (red) in control, siCD93-treated, and siMMRN2-treated HDBECs cultured under confluent conditions on gelatin-coated surfaces. Scale bars: 20 μm. (**E**) Real-time qPCR for fibronectin in control (Mock and siCtrl), CD93-downregulated (siCD93_1 and siCD93_2), and MMRN2-silenced cells (siMMRN2_3 and siMMRN2_6). Values represent mean ± SEM (3 independent experiments); 1-way ANOVA with Dunnett’s multiple-comparisons test. (**F**) Fibronectin (green) and F-actin (red) in brain endothelial cells isolated from WT and CD93^–/–^ mice and cultured on gelatin-coated surfaces. Scale bars: 20 μm. (**G**) Rescue of fibronectin fibrillogenesis disrupted by CD93 downregulation. Immunofluorescent staining for fibronectin (green) and F-actin (red) in HDMECs stably expressing WT-CD93 insensitive to siRNA knockdown (WT-CD93 HDMEC) or untransfected cells (Control HDMEC) after endogenous CD93 silencing (siCD93_1 and siCD93_2). Scale bars: 20 μm. (**H**) Rescue of fibronectin fibrillogenesis upon MMRN2 downregulation (siMMRN2) in the presence or absence of conditioned medium derived from control cells (+Mock CM or +siCtrl CM). Immunofluorescent staining for fibronectin (green) and F-actin (red). Scale bars: 20 μm.

Costaining of CD93 and MMRN2 in confluent endothelial cells showed colocalization of these proteins along fibrillar structures distributed on the endothelial monolayer (Figure 6A). CD93 did not colocalize with filamentous F-actin or other cytoskeletal proteins, including tubulin or vimentin, in confluent endothelial cells (Supplemental Figure 6, A–C). Instead, a deeper characterization of the subcellular distribution of CD93 and MMRN2 revealed that both proteins colocalize with fibronectin fibers (siCtrl in Figure 6, B and C). The colocalization between CD93 and fibronectin was dramatically inhibited in MMRN2-silenced endothelial cells (siMMRN2_3 in Figure 6B). In contrast, downregulation of CD93 did not affect the colocalization between MMRN2 and fibronectin fibers (siCD93_1 in Figure 6C). These results indicate that MMRN2 is required for CD93 interaction with fibronectin in extracellular matrix.

Costaining with vesicular markers revealed that CD93 colocalized with caveolin-1–coated vesicles in endothelial cells, while MMRN2 was found partially associated with vWF-positive vesicles (Supplemental Figure 6, D and E). Our data suggest that the interaction between CD93 and MMRN2 occurs in the cell membrane–extracellular matrix interface.

Fibronectin is a major component of the extracellular matrix and is essential for sprouting angiogenesis and cell migration (19). Therefore, we investigated whether the interaction of CD93 with MMRN2 affects the organization of fibronectin fibrils. Control endothelial cells grown on gelatin formed a dense fibronectin matrix and a well-organized fibrillar network (Mock and siCtrl in Figure 6D). However, downregulation of either CD93 or MMRN2 in endothelial cells induced a disruption in the fibronectin fibrillar network as compared with controls (siCD93 and siMMRN2 in Figure 6D). In contrast, fibronectin gene expression in siCD93 and siMMRN2 cells was similar to that in controls (Figure 6E). These results indicate that CD93 and MMRN2 are important for the organization of fibronectin into fibrillary structures, but that they do not regulate fibronectin expression in endothelial cells. Consistent with these results, decreased fibronectin fibrillogenesis was observed in brain endothelial cells isolated from CD93^–/–^ mice compared with those isolated from WT mice (Figure 6F). Disrupted fibronectin fibrillogenesis was restored by lentiviral transfection with a WT CD93 construct in endothelial cells silenced for endogenous CD93 (Figure 6G). A similar rescue effect on fibronectin fibrillogenesis was observed when siMMRN2 cells were cultured in conditioned medium derived from control cells (Figure 6H). These data point out a novel role of MMRN2 and CD93 interaction in regulating organization of fibronectin in extracellular matrix.

### Knockdown of CD93 or MMRN2 impairs β_1_ integrin activation and phosphorylation of focal adhesion kinase in endothelial cells.

**Figure 7:**
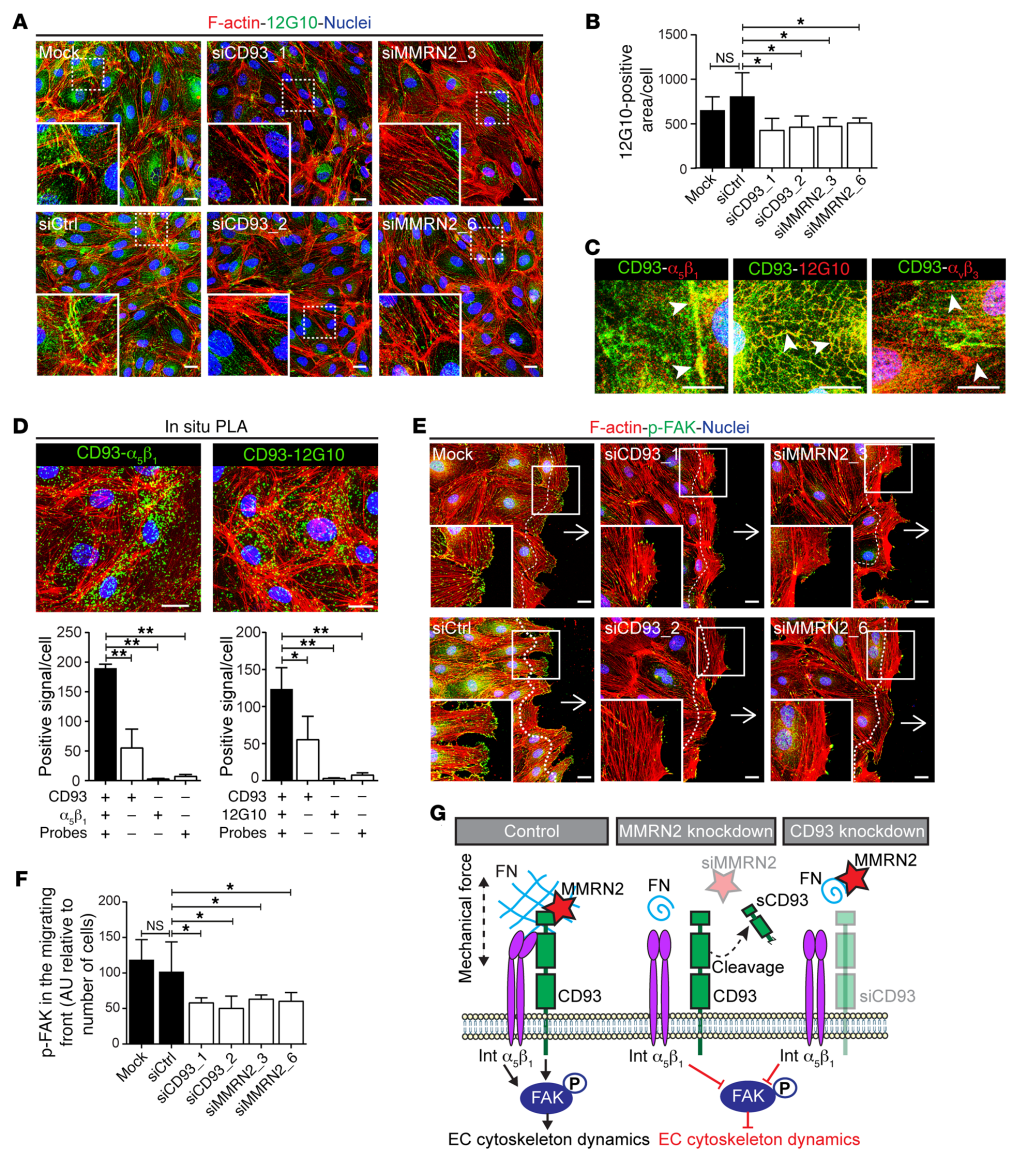
CD93 and MMRN2 interact with α_5_β_1_ integrin in endothelial cells and regulate its activity. (**A**) Immunofluorescent staining of the active β_1_ integrin (12G10) in control, CD93-silenced (siCD93_1, siCD93_2), and MMRN2-silenced (siMMRN2_3, siMMRN2_6) HDBECs cultured under confluent conditions. Scale bars: 20 μm. (**B**) Quantification of 12G10 staining in control, siCD93, and siMMRN2 cells. Values represent mean ± SEM expressed as arbitrary units (AU) of 12G10-positive area normalized by cell number (*n* = 8 different areas of the cell monolayer). *P < 0.05. (**C**) Colocalization between CD93 and α_5_β_1_ integrin and active β_1_ (12G10) is shown by arrowheads. Different distribution of CD93 and α_v_β_3_ integrin is shown by arrowheads. Scale bars: 20 μm. (**D**) In situ PLA for CD93 and α_5_β_1_ integrin as well as CD93 and active β_1_ integrin (12G10) in HDBECs. Positive signal (green dots) indicates proximity between CD93 and integrins. F-actin (red) and nuclei (blue). Scale bars: 20 μm. Bars represent the quantification of CD93 and α_5_β_1_ integrin or CD93 and active β_1_ integrin interaction based on the number of positive signals per cell (*n* = 3 independent experiments). *P < 0.05; **P < 0.01. (**E**) Y397-phosphorylated FAK staining (p-FAK) in the migrating front of control or siCD93- and siMMRN2-silenced cells at 6 hours after scratch wound. Arrows indicate the direction of migration. Scale bars: 20 μm. (**F**) Quantification of the p-FAK–positive signal along the migrating front (20 μm distance from the migrating front, indicated by dotted line in **E**). Bars represent arbitrary units (AU) of p-FAK signal normalized by the number of cells at the migrating edge. *P < 0.05. Values represent mean ± SEM (4 different areas along the migrating edge, 3 independent experiments). 1-Way ANOVA with Dunnett’s post-test was used in all graphs. (**G**) Schematic representation of CD93 and MMRN2 interacting complex. FB, fibronectin; MMRN2, multimerin-2; sCD93, soluble CD93; Int α_5_β_1_, α_5_β_1_ integrin; p-FAK, phosphorylated focal adhesion kinase.

Integrins are transmembrane proteins with a well-described function in the regulation of cell-matrix adhesion, extracellular matrix organization, and cell migration (20). In particular, α_5_β_1_ integrin plays a key role in the regulation of fibronectin fibrillogenesis and extracellular matrix deposition (21). We therefore investigated whether β_1_ integrin activity was altered in CD93- or MMRN2-downregulated cells. β_1_ Integrin activation was assessed by immunofluorescent staining using an antibody that specifically recognizes the active conformation of β_1_ integrin (clone 12G10). A striking reduction of active β_1_ integrin was observed in endothelial cells when either CD93 or MMRN2 was silenced (Figure 7, A and B). This reduction in β_1_ integrin activation was confirmed by Western blot analysis, indicating that CD93 and MMRN2 affect integrin activation rather than expression (Supplemental Figure 7A). The important role of CD93 in regulating β_1_ integrin activation was confirmed in brain endothelial cells isolated from CD93^–/–^ and WT mice, showing a striking reduction in β_1_ integrin activation in the absence of CD93 (Supplemental Figure 7B).

β_1_ Integrin can interact with several integrin α subunits in endothelial cells, and the main receptor for fibronectin is the α_5_β_1_ integrin. CD93 colocalized with α_5_β_1_ integrin in confluent HDBECs and in filopodia of sparsely seeded endothelial cells (arrowheads in Figure 7C and Supplemental Figure 7C). Costaining with the 12G10 antibody revealed colocalization between CD93 and activated β_1_ integrin in endothelial cells. α_v_β_3_ Integrin can also bind to fibronectin in endothelial cells, but we did not observe colocalization between CD93 and α_v_β_3_ integrin (Figure 7C). The CD93-α_5_β_1_ interaction was further confirmed by PLA showing statistically significant interaction between CD93 and α_5_β_1_ as well as CD93 and active β_1_ integrin (Figure 7D).

Focal adhesion kinase (FAK) is activated downstream of integrin activation and plays a key role in focal adhesion turnover as well as cytoskeleton organization and cell migration (22). In line with a requirement of CD93 and MMRN2 for full β_1_ integrin activation, we observed reduced FAK activation, monitored as Y397-phosphorylated FAK (p-FAK), in endothelial cells treated with siRNA targeting either CD93 or MMRN2. We detected high levels of p-FAK in the focal adhesion sites at the leading edge of migrating control endothelial cells, while a striking reduction of p-FAK levels were found in the migrating front of endothelial cells silenced for either CD93 or MMRN2 (Figure 7E, quantified in Figure 7F).

These data indicate that CD93 interacts with α_5_β_1_ integrin and modulates its activation, leading to the phosphorylation of the downstream molecule FAK, and inducing fibronectin fibrillogenesis required for cell migration. The extracellular matrix protein MMRN2 stabilizes CD93 and thereby enhances the activation of α_5_β_1_ integrin. The proposed mechanism through which the CD93-MMRN2 complex interacts with integrins and fibronectin, thus affecting endothelial cell dynamics, is depicted in Figure 7G.

**Figure 8:**
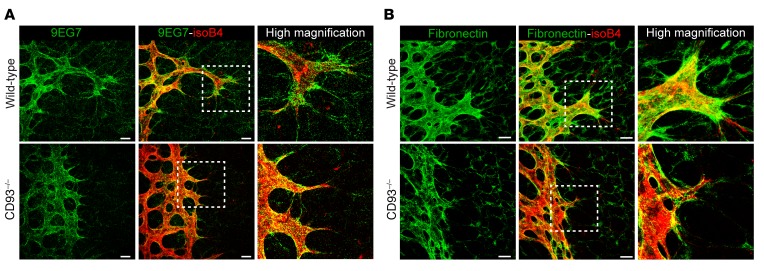
CD93-knockout mice show reduced β_1_ integrin activation and disrupted fibronectin matrix during retina angiogenesis. (**A**) Immunofluorescent staining of active β_1_ integrin (9EG7) in WT and CD93^–/–^ P6 mouse retina. The vasculature is visualized by isolectin B4. Scale bars: 20 μm. High-magnification images show inhibition of active β_1_ integrin in the filopodia of CD93^–/–^ compared with WT mouse retina. (**B**) Immunofluorescent staining of fibronectin and isolectin B4 in WT and CD93^–/–^ P6 mouse retina. Scale bars: 20 μm. High-magnification image shows a disruption of the fibronectin matrix in the proximity of endothelium and filopodia in CD93^–/–^ compared with WT mouse retina.

β_1_ Integrin is critical for vessel growth and maturation and is indispensable for efficient endothelial sprout formation (23). Given the significant inhibition in endothelial sprouts and endothelial filopodia observed in CD93^–/–^ retinas (Figure 2, D and E), integrin activation and fibronectin fibrillogenesis was assessed during developmental angiogenesis in P6 WT and CD93^–/–^ mouse retinas. Consistent with a key role of CD93 in β_1_ integrin activation, a dramatic inhibition of the active form of β_1_ integrin (recognized by the 9EG7 antibody) was observed in the sprouting front and filopodia of CD93^–/–^ retinas compared with WT mice (Figure 8A). In addition, fibronectin staining showed disruption of fibronectin fibers in the sprouting front and filopodia of CD93^–/–^ retinas (Figure 8B).

### CD93 and MMRN2 expression correlates with increased fibronectin deposition in human glioma vessels.

**Figure 9:**
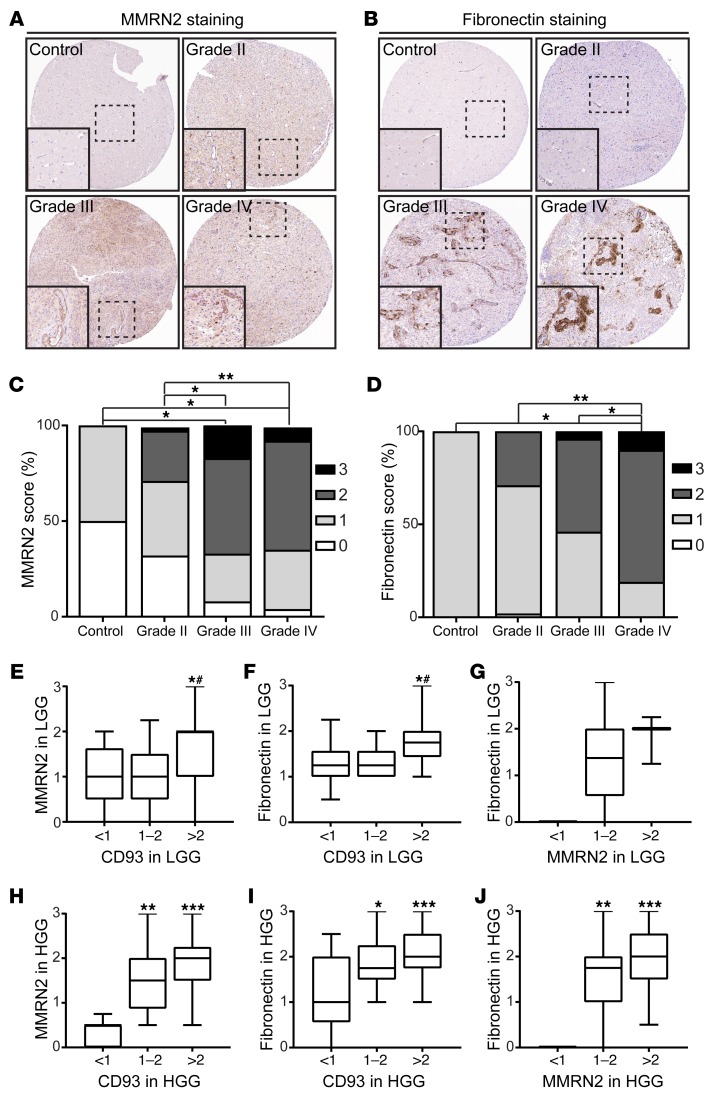
MMRN2 and fibronectin expression is increased in human high-grade glioma. (**A** and **B**) Immunohistochemical staining of MMRN2 (**A**) and fibronectin (**B**) in nontumor control brain and WHO grade II, grade III, and grade IV human glioma tissue. (**C** and **D**) Semiquantitative scoring of the fraction of MMRN2-positive (**C**) and fibronectin-positive (**D**) blood vessels in human glioma tissue microarrays. The frequency of positively staining vessels in 235 samples from human glioma and nontumor control brain was scored in a blinded fashion on a scale from 0 to 3 (0, no vessels stained; 1, minority of vessels stained; 2, majority of vessels stained; 3 strong staining in the majority of vessels). Results were averaged and plotted as percentage of samples per score in nontumor control brain (2 biopsies), WHO grade II (65 biopsies), grade III (29 biopsies), and grade IV samples (69 biopsies); *P < 0.005, **P < 0.0001; 1-way ANOVA with Tukey’s post-test. (**E**–**G**) Comparison between scoring of MMRN2 and CD93 (**E**), of fibronectin and CD93 (**F**), and of fibronectin and MMRN2 (**G**) in low-grade glioma (LGG, WHO grade II). *P < 0.05 vs. <1 score; ^#^*P* < 0.05 vs. 1–2 score; 1-way ANOVA with Tukey’s post-test. Comparison between scoring of MMRN2 and CD93 (**H**), of fibronectin and CD93 (**I**), and of fibronectin and MMRN2 (**J**) in high-grade glioma (HGG, WHO grade III–IV). *P < 0.05, **P < 0.005, ***P < 0.0001 vs. <1 score; 1-way ANOVA with Tukey’s post-test.

Fibronectin fibrillogenesis is a known feature of tumor angiogenesis. To determine whether CD93 is coexpressed with MMRN2 and fibronectin fibrils in human glioma vessels, we analyzed the fraction of blood vessels positive for MMRN2 (Figure 9A) and fibronectin (Figure 9B) in glioma tissue microarrays, containing a total of 235 samples of human control brain and WHO grade II–IV glioma tissues, and correlated the scores with our previous scoring of CD93 expression (11). Since MMRN2 and fibronectin were heterogeneously expressed in tumor vessels, a semiquantitative scoring method was used based on the proportion of positive vessels in each core. Similar to our previous observation that CD93 expression is increased in the vasculature of human high-grade glioma (HGG) (11), MMRN2 and fibronectin were significantly elevated in WHO grade III and IV glioma compared with low-grade glioma (LGG; WHO grade II) or control brain samples (Figure 9, C and D). When correlating the scoring of MMRN2 staining with the scoring of CD93 staining in the same tissue microarray cores, we found that high levels of CD93 corresponded to significantly higher levels of MMRN2 in samples from LGG (Figure 9E) and HGG (Figure 9H). Similar results were obtained when we correlated fibronectin scores with CD93 scores in the vessels of LGG (Figure 9F) and HGG (Figure 9I). HGG samples with high levels of vascular MMRN2 showed significantly higher levels of fibronectin associated with the vessels (Figure 9J). No significant association was found between fibronectin and MMRN2 in LGG (Figure 9G). These results indicate that CD93, fibronectin, and MMRN2 are coexpressed in tumor vessels in glioma, and that the levels of these proteins are increased in higher malignancy grades, suggesting a functional role of the CD93-MMRN2-fibronectin complex during tumor angiogenesis.

### CD93 is essential for β_1_ integrin activation and fibronectin fibrillogenesis in GL261 tumor vessels.

**Figure 10:**
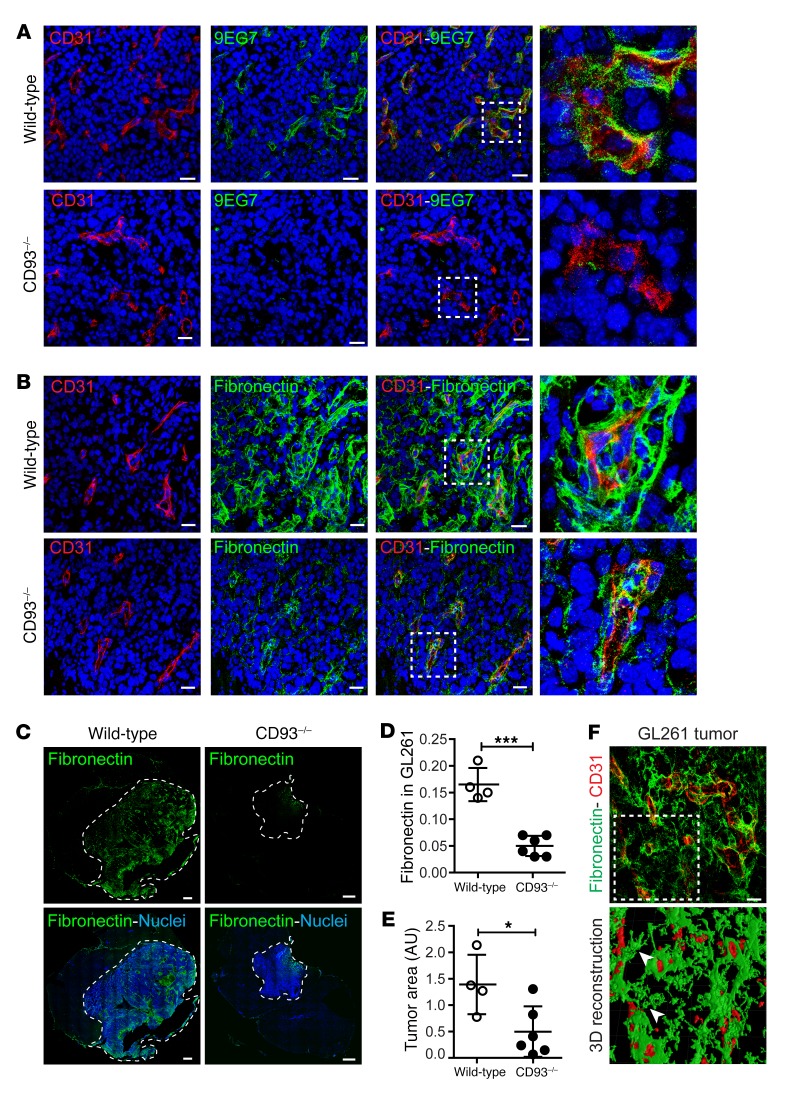
CD93 deficiency results in diminished β_1_ integrin activation and fibronectin deposition in GL261 vasculature. (**A**) Immunofluorescent staining of the active conformation of β_1_ integrin (9EG7) in GL261 tumor vasculature of WT and CD93^–/–^ mice. CD31 was used as vascular marker (red) and Hoechst as nuclear marker (blue). High-magnification images show the levels of 9EG7 staining associated with the vasculature in WT and CD93^–/–^ mice. Scale bars: 20 μm. (**B**) Immunofluorescent staining of fibronectin and CD31 in GL261 tumor vasculature of WT and CD93^–/–^ mice. High-magnification images show fibronectin associated with the vasculature in WT and CD93^–/–^ mice. Scale bars: 20 μm. (**C**) Tile scan of GL261 tumor in WT and CD93^–/–^ mice stained for fibronectin. Scale bars: 500 μm. (**D**) Quantification of fibronectin-positive signal in WT (*n* = 4) and CD93^–/–^ (*n* = 6) mice. Values represent mean ± SEM expressed as arbitrary units (AU) of fibronectin-positive area normalized by total tumor area. ***P < 0.0001; 2-tailed *t* test. (**E**) Quantification of tumor area in WT (*n* = 4) and CD93^–/–^ (*n* = 6) mice. Values represent mean ± SEM expressed as AU of the total tumor area determined by nuclei staining. *P < 0.05; 2-tailed *t* test. (**F**) 3D volume reconstruction of fibronectin (green) and CD31 (red) immunofluorescent staining in a GL261 vibratome section. Arrowheads indicate fibronectin fibers associated with vessels (CD31 positive). Scale bar: 20 μm.

To determine whether CD93 is required for efficient β_1_ integrin activation and fibronectin fibrillogenesis during tumor angiogenesis, we analyzed tumor vessels in intracranial GL261 gliomas in WT and CD93^–/–^ mice. By immunofluorescence staining using an antibody that specifically recognizes the active form of β_1_ integrin (clone 9EG7), we found that β_1_ integrin was highly activated in tumor vessels (Wild-type in Figure 10A). In contrast, a dramatic reduction of β_1_ integrin activation was observed in GL261 tumor vessels in CD93^–/–^ mice (CD93^–/–^ in Figure 10A). In normal vessels adjacent to the tumor site, β_1_ integrin activation was low or absent in both WT and CD93^–/–^ mice (Supplemental Figure 8A).

Immunostaining revealed an abundant deposition of fibronectin in the GL261 tumor tissue in WT mice, predominantly associated with the tumor vessels (Figure 10B). Notably, a substantial reduction of fibronectin deposition was found in the CD93^–/–^ GL261 tumor, and disrupted fibronectin fibers were observed associated with the tumor vasculature (Figure 10B). Quantification of the fibronectin signal revealed a significant reduction of fibronectin deposition in GL261 tumors from CD93^–/–^ mice compared with tumors from WT mice (Figure 10, C and D). No obvious differences in fibronectin deposition were found in the normal vasculature of CD93^–/–^ mice compared with WT mice, and fibronectin levels in normal vessels were considerably lower than those in tumor vessels (Supplemental Figure 8B). Fibronectin gene expression analysis showed a significant upregulation of fibronectin in tumor tissue as compared with nontumor brain tissue, and no differences were found when WT and CD93^–/–^ mice were compared (Supplemental Figure 8C). Fibronectin expression was considerably higher in brain endothelial cells than in GL261 cells, suggesting a main contribution of the vasculature in fibronectin production in this tumor model (Supplemental Figure 8D). Consistent with our previous report, the mean GL261 tumor area was significantly reduced in CD93^–/–^ mice as compared with WT mice (Figure 10E and ref. 11). These results indicate a nonredundant role of CD93 in promoting β_1_ integrin activation and fibronectin fibrillogenesis during glioma angiogenesis. A 3D volume reconstruction of fibronectin deposition and CD31-positive vessels in GL261 tumors revealed that fibronectin fibrils are consistently associated with blood vessels (arrowheads Figure 10F). Thus, our data show that fibronectin matrix is mainly deposited and organized in fibrillary structures by endothelial cells in GL261 tumors, and indicate that CD93 expression is critical for correct organization of fibronectin during tumor angiogenesis.

## Discussion

CD93 is a member of group XIV in the C-type lectin superfamily, which also includes thrombomodulin, CLEC14A, and endosialin, all of which are highly expressed in tumor vessels (24–26). While CD93 and CLEC14A are expressed in tumor endothelial cells, endosialin is mainly expressed in pericytes (11, 24, 27). Notably, CD93, along with CLEC14A, was recently included as one of the top 20 genes in a common angiogenesis signature of human tumors (28). Because of a high expression of group XIV C-type lectins in human cancer during tumor angiogenesis, these proteins have emerged as potential targets for therapy. However, little is known regarding the mechanisms through which they regulate vessel formation in cancer.

MMRN2 is an endothelial cell–specific member of the EMILIN family, secreted homotrimeric glycoproteins that assemble into multimers in the extracellular matrix (29). MMRN2 was previously found to be upregulated in tumor vessels in experimental cancer models, and to bind to CLEC14A (30, 31). Our study shows that MMRN2 is a key interaction partner of CD93 during tumor angiogenesis. We found that MMRN2 expression correlates to WHO grade in glioma, and that the level is considerably lower in normal brain vessels. Gliomas with high vascular expression of CD93 express higher levels of MMRN2 and show enhanced fibronectin deposition. While the manuscript of this article was being finalized, the CD93-MMRN2 interaction was confirmed by 2 studies demonstrating direct binding of MMRN2 with CD93 and endosialin (32, 33). Interestingly, CD93 and CLEC14A interact with the same part of MMRN2, suggesting that CD93 and CLEC14A may compete for binding. Shedding of CLEC14A from the endothelial cell surface catalyzed by rhomboid-like 2 protein is known to inhibit migration of endothelial cells (34). We show that MMRN2 protects CD93 from cleavage of its extracellular domain. The protease responsible for shedding of CD93 in endothelial cells has not yet been identified. In human myeloid cells CD93 is cleaved by MMPs (35). Similarly, MMRN2 can be processed by MMP-9, which has been suggested to regulate endothelial sprouting (36). Fibronectin was not found to directly interact with CD93 in our coimmunoprecipitation/mass spectrometry analysis. Our data are consistent with CD93 binding to MMRN2 during formation of the angiogenic sprout, enabling its interaction with fibronectin and subsequent fibrillogenesis as further discussed below. Our data demonstrate a nonredundant role of CD93 in orchestrating integrin activation and fibronectin organization during tumor angiogenesis. However, the common binding to MMRN2 by proteins of the group XIV C-type lectin superfamily indicates a network of coregulation and mutual interaction during angiogenesis, which is an interesting area for further study.

In the embryoid body model of vascular development, CD93 was exclusively expressed in tip cells. Tip cells are specialized nonproliferating endothelial cells that form numerous filopodia and guide endothelial cell sprouting during angiogenesis (37). Tip cell differentiation is dependent on VEGF/VEGFR and Notch signaling pathways (38, 39). Several tip cell markers, including VEGFR2, endocan/ESM1, and angiopoietin 2, are generally upregulated in tumor vessels and were identified in our analysis of genes associated with vascular abnormality in glioblastoma (7, 40, 41). The enrichment of tip cell–associated genes in tumor vessels may be connected to a high expression of VEGF in the tumor microenvironment, leading to perturbed tip and stalk cell conversion (42). Indeed, we have previously found that pleiotrophin promotes vascular abnormalities associated with an exceptionally high perivascular deposition of VEGF (8). Targeting molecules important for tip/stalk conversion may affect angiogenesis. In CD93^–/–^ mice, there are fewer filopodia in the sprouting front during vascular development in the retina, suggesting that CD93 is important for tip cell behavior. However, we did not detect any difference in the sprouting distance. This may suggest either that there is a surplus of filopodia formed in the retina, or that compensatory mechanisms rescue endothelial sprouting despite decreased filopodia formation. Nevertheless, our data are consistent with an important role of CD93 in filopodia formation during angiogenesis.

β_1_ Integrin is important for filopodia formation, and basal filopodia have recently been shown to regulate organization of fibronectin into pillars between germ layers (43). We found that CD93 colocalized with α_5_β_1_ integrins in endothelial filopodia and identified CD93 as a novel regulator of β_1_ integrin activation during tumor angiogenesis. Knockdown of either CD93 or MMRN2 led to a striking decrease in β_1_ integrin activation in endothelial cells in vitro. Similarly, CD93 deficiency reduced β_1_ integrin activation in murine GL261 glioma tumor vessels in vivo. Inhibition of integrin activation decreases angiogenesis and reduces tumor growth in several models (44, 45), consistent with our observation of deficient integrin activation and decreased tumor growth observed in CD93^–/–^ mice. Fibronectin-binding integrins are required for assembly of soluble fibronectin into fibers, and α_5_β_1_ integrin is the main integrin serving this function (21, 46, 47). Consistent with an important role of α_5_β_1_ integrin in fibronectin fibrillogenesis, siRNA knockdown or deficiency of either CD93 or MMRN2 in endothelial cells resulted in disruption of the fibronectin fibrillar network formation in vitro. In vivo, the fibronectin fibrillary network associated with the vasculature was disrupted during tumor angiogenesis in GL261 gliomas implanted intracranially in CD93^–/–^ mice. Fibronectin fibrillogenesis promotes vascular maturation during brain angiogenesis, which may explain the loss of vascular integrity and functionality in GL261 tumors noted in CD93^–/–^ mice (11). This suggests that inhibition of the interaction between CD93 and MMRN2 may lead to disruption of vascular integrity in tumors, representing a new target for pharmaceutical intervention. Above all, we have uncovered a novel CD93/MMRN2/β_1_ integrin/fibronectin signaling axis that orchestrates tumor angiogenesis and vascular stability in cancer.

## Methods

Detailed information is given in the Supplemental Methods.

### Tumor cell and primary endothelial cell culture.

GL261 glioma cells (a gift from Geza Safrany, National Research Institute for Radiobiology and Radiohygiene, Budapest, Hungary) were cultured in DMEM (Life Technologies) supplemented with 10% FBS (Sigma-Aldrich) at 37°C and 5% CO_2_/95% air in a humidified chamber.

Human dermal microvascular endothelial cells (HDMECs) or human dermal blood endothelial cells (HDBECs) (PromoCell) were cultured in gelatin-coated culture dishes in Endothelial Cell Basal Medium with full supplements (PromoCell, EBM-MV2) at 37°C and 5% CO_2_/95% air in a humidified chamber.

Primary mouse brain endothelial cells were isolated from 12-week-old C57BL/6 WT or CD93^–/–^ mice as previously described (48).

Mycoplasma test was routinely performed in all cell cultures used in this study.

### Mice.

CD93^–/–^ mice (49) and heterozygous and WT littermates were bred in house. C57BL/6 WT mice were purchased from Taconic M&B.

### Coimmunoprecipitation and mass spectrometry analysis.

Coimmunoprecipitation to identify CD93-interacting proteins was performed using the Pierce coimmunoprecipitation kit (Thermo Fisher Scientific) according to the manufacturer’s instructions. Briefly, total protein extract obtained from subconfluent HDMECs was immunoprecipitated with mouse anti–human CD93 antibody (MBL Life Science, D198-3) or negative control mouse IgG (Santa Cruz Biotechnology, sc-2025). CD93 coimmunoprecipitated samples and IgG negative controls of 5 independent experiments were analyzed by mass spectrometry for protein identification by LC-Orbitrap MS/MS at the Mass Spectrometry–Based Proteomics Facility, Science for Life Laboratory (SciLifeLab), Uppsala University. Protein identifications were obtained by a database search using the quantitation software MaxQuant 1.5.1.2.

### Proximity ligation assay.

PLA was performed using the Duolink II kit (Sigma-Aldrich, DUO92104) according to a standard protocol at the PLA Proteomics Facility, SciLifeLab, Uppsala University. Briefly, HDBECs were incubated with mouse anti–human CD93 (MBL Life Science, D198-3) and goat anti–human MMRN2 (Santa Cruz Biotechnology, sc54021) primary antibodies or with rabbit anti–human CD93 (HPA, HPA009300) and mouse anti–human β_1_ integrin (12G10, Abcam, ab30394) or mouse anti–human α_5_β_1_ integrin (clone JBS5, Millipore, mab1969) primary antibodies. Cells were then incubated with specific PLA secondary probes. Actin and nuclei were detected by Alexa Fluor 488–phalloidin and Hoechst, respectively. Single primary antibodies with PLA probes and PLA probes without primary antibodies were included as negative controls. The PLA signal was quantified in 5 different areas of the cell monolayer using Duolink ImageTool software.

### siRNA transfections.

HDBECs were incubated with scrambled control siRNA or siRNA to CD93 (Hs_CD93_1 and Hs_CD93_2) or siRNA to MMRN2 (Hs_MMRN2_3 and Hs_MMRN2_6) (FlexiTube, Qiagen) at a concentration of 2 nM in a mixture of 20% Opti-MEM (Life Technologies) in endothelial cell medium supplemented with 30 μl/ml Lipofectamine RNAiMAX (Life Technologies) for 4–6 hours, after which the medium was replaced with fresh medium. Experiments were performed at day 2–3 after siRNA transfection.

### Lentivirus production.

For knockdown of CD93 or MMRN2, shRNA sequences targeting either of the genes were subcloned under the control of the H1 promoter into pBMN (EF1a-GFP-Puro) vectors. The shRNA sequence targeting CD93 is CAGGATTGTGTCAACACTCTA, and the sequence targeting MMRN2 is CAGCTTGTATCAGACATGGTA. This vector contains GFP for visualization and the puromycin resistance gene as a selection marker. Lentivirus particles were produced as previously described (50).

Generation of CD93-WT HDMECs was performed as previously described (11). Briefly, cDNA sequences for human CD93 (WT-CD93) were designed and purchased from GenScript. These sequences contain silent mutations in the regions where the CD93 siRNA sequences used in this study bind. The sequences were subcloned into a third-generation self-inactivating lentiviral vector together with coding sequences for GFP and puromycin to generate pBMN (CD93 WT).

### Western blot.

HDBECs were lysed in NuPAGE LDS Sample buffer under reducing or nonreducing conditions, according to the primary antibody manufacturer’s recommendations. The samples were separated onto a 4%–12% Bis-Tris gel (Life Technologies) and blotted to a nitrocellulose membrane (Immobilon). To analyze proteins secreted or cleaved from endothelial cells, conditioned medium derived from HDBECs maintained for 24 hours in EBM media supplemented with 1% FBS was collected, cell debris were removed by centrifugation, and 25 μg of total protein was separated onto a gel. The membranes were blocked and probed with anti–human CD93 (HPA, HPA009300), anti–human β_1_ integrin (K20, Santa Cruz Biotechnology, sc18887), anti–human CD93 (MBL Life Science, D198-3), anti–human MMRN2 (Santa Cruz Biotechnology, sc54021), or anti–human β_1_ integrin (12G10, Abcam, ab30394) or with anti–human β-actin (Santa Cruz Biotechnology, sc-1615) followed by appropriate horseradish peroxidase–labeled secondary antibodies. A representative blot image of at least 3 independent experiments is shown.

### RNA extraction and quantitative PCR.

RNA from control or siRNA-treated endothelial cells as well as tumor and nontumor brain tissue was extracted using the RNeasy Plus Mini Kit (Qiagen). Total RNA was transcribed using Superscript III reverse transcriptase in 20 μl total volume containing 250 ng of random hexamers and 40 units of RNase OUT inhibitor (Life Technologies). mRNA expression of CD93, MMRN2, and fibronectin was quantified relative to HPRT by real-time PCR in duplicate reactions per sample with 0.25 μM forward and reverse primer in SYBR Green PCR Master Mix (Life Technologies). Primer sequences are listed in Supplemental Methods.

### Inhibitors.

Inhibition of de novo protein synthesis of control or siMMRN2-transfected cells was performed using 50 μg/ml cycloheximide (Sigma Aldrich, 66-81-9). Cells were lysed at different time points after treatment (30, 60, and 240 minutes). Proteasome inhibition was performed using 20 μM lactacystin during 8 hours in control and siMMRN2-transfected cells. Inhibition of lysosomal protein degradation was performed using 10 mM NH_4_Cl during a period time of 18 hours in control and siMMRN2-transfected cells. Subsequently, cells were harvested, and cellular CD93 protein stability was assessed by Western blot analysis.

### Migration assay.

A double-sided wound was produced on a confluent HDBEC monolayer and maintained in EBM supplemented with 10% FBS. At 2, 4, and 6 hours after the scratch, images were taken of 4 different fields along the scratch, covering more than 90% of the initial cell-depleted area. The cell-free area for each field was calculated using ImageJ software (NIH), and cell migration (wound closure) was expressed as a percentage of the cell-free area at time point 0. Alternatively, at 6 or 8 hours after wounding, the cells were fixed for immunolabeling and confocal microscopy analysis. Rescue experiments were performed using 2-well culture inserts (ibidi, 80209). A representative image of each condition in at least 3 independent experiments is shown.

### Embryoid body culture.

Mouse embryonic stem (ES) cell culture and formation of embryoid bodies (EBs) was performed as previously described (51) and detailed in Supplemental Methods. Briefly, cultured ES cells were aggregated in hanging drops in order to form EBs and subsequently placed on 8-well chamber slides (2D EB culture) or between 2 layers of a collagen I gel (3D EB culture). EBs were cultured in the presence of 30 ng/ml VEGF-A165. Medium was changed every other day, and, at day 14, EBs were fixed in 4% paraformaldehyde and processed for whole-mount immunofluorescence. A representative image of at least 5 individual EBs is shown. In specific experiments, ES cells were transduced with lentiviruses with CD93 or MMRN2 shRNA construct or empty vector as control. GFP-positive cells were isolated by FACS. Transduced ES cells were cultured as described above.

### Immunofluorescent staining of endothelial cells.

siRNA-transfected HDBECs or mouse brain endothelial cells were stained with anti-CD93 (MBL Life Science, D198-3), anti-CD93 (HPA, HPA009300), anti-MMRN2 (Santa Cruz Biotechnology, sc54021), anti-fibronectin (Abcam, ab2413), anti-fibronectin (Sigma-Aldrich, F7387), anti–β_1_ integrin (12G10, Abcam, ab30394), anti–α_5_β_1_ integrin (clone JBS5, Millipore, mab1969), anti–α_v_β_3_ integrin (Millipore, MAB1976), anti–p-FAK (Y397, Abcam, ab39967), anti–α-tubulin (Abcam, ab52866), anti-vimentin (Abcam, ab92547), anti-vWF (Dako, A0082), and anti–caveolin-1 (Cell Signaling Technology, CST3238). Cells were subsequently incubated with appropriate Alexa Fluor–conjugated secondary antibodies (Invitrogen). Nuclei and cytoskeleton were visualized by Hoechst and Alexa Fluor 647–labeled or Texas Red–conjugated phalloidin, respectively (all from Life Technologies). Cells were analyzed under a confocal microscope (Leica SP8).

### Immunofluorescent staining of tumor sections, retina, and EBs.

Cryosections of human grade IV glioma and murine GL261 glioma sections were stained with anti-CD31 (2H8, Thermo Fisher Scientific, MA3105), anti-CD93 (R&D Systems, AF1696), anti-MMRN2 (MyBioSource, MBS2028221), anti-fibronectin (Abcam, ab2413), and anti–β_1_ integrin (9EG7, BD Pharmingen, 553715). Sections were washed and incubated with Alexa Fluor–conjugated secondary antibodies (Invitrogen). Nuclei were visualized by Hoechst (Sigma-Aldrich). A representative image of at least 10 individual immunofluorescence stainings is shown.

To analyze and quantify the retina vasculature, CD93^–/–^ and WT as well as heterozygous littermate eyes (P6) were stained with anti-CD31 (2H8, Thermo Fisher Scientific, MA3105) or Alexa Fluor 488–conjugated isolectin B4 (Thermo Fisher Scientific, I21411) as well as anti-ERG (Abcam, ab92513), anti-CD93 (R&D Systems, AF1696), anti-MMRN2 (MyBioSource, MBS2028221), anti-fibronectin (Abcam, ab2413), and anti–β_1_ integrin (9EG7, BD Pharmingen, 553715) primary antibodies followed by incubation with Alexa Fluor–conjugated secondary antibodies (Invitrogen). A representative image of at least 10 individual retina immunofluorescent stainings is shown.

For whole-mount EB immunostaining, EBs were probed with the following primary antibodies: anti-CD31 (2H8, Thermo Fisher Scientific, MA3105), anti-CD93 (R&D Systems, AF1696), and anti-MMRN2 (MyBioSource, MBS2028221). Then EBs were incubated with appropriate fluorescently labeled secondary antibodies and analyzed under a confocal microscope (Leica SP8) or fluorescent microscope (Leica DMi8).

### Tissue microarray and image analysis.

Vascular expression of MMRN2 and fibronectin was scored in tissue microarrays containing duplicate tissue cores per sample (1 mm diameter) of 235 samples of human low-grade (WHO grade II, *n* = 65) and high-grade (WHO grade III, *n* = 29; WHO grade IV, *n* = 69) gliomas of various histologic subtypes, and nontumor brain tissue used as control (*n* = 2). Immunohistochemical staining was performed as previously described (52) using antibodies recognizing MMRN2 (HPA020741, Atlas Antibodies) and fibronectin (HPA027066, Atlas Antibodies) with DAB as a substrate. The frequency of positively stained vessels was scored in a blinded fashion by 2 researchers individually on a scale from 0 to 3 (0 = no vessels stained, 1 = minority of vessels stained, 2 = majority of vessels stained, 3 = strong staining in the majority of vessels). Data are shown as average of 2 individual scorings.

### Image analysis.

Image analysis was performed using ImageJ software. For the quantification of retinal parameters, migration of vessels was analyzed by measurement of the radial distance from the optic nerve head to the vascular front at the retinal periphery in at least 4 mice per genotype. The number of filopodia was counted per 100 μm of leading endothelial vessel membrane under ×63 magnification confocal micrographs. The sprouting front length was measured from the tip to the base of the sprout in at least 4 randomly selected front areas per retina and at least 4 mice per genotype; values represent the relative sprout length normalized to the migrating front length of each field used in the analysis. For the quantification of filopodia formation in control or MMRN2- and CD93-silenced endothelial cells, filopodia were counted in at least 5 randomly selected images with at least 5 single cells per condition. For the CD93 quantification at the EC migrating front upon MMRN2 knockdown, a minimum of 4 images of the migrating front area per condition were used, and CD93 localization was analyzed by application of a threshold along the migrating edge determined by the nuclei of the cells at the leading edge and the migrating front in the wound healing assay. Similarly, p-FAK was quantified in the migrating front area (20 μm at the leading edge) in control and siCD93- or siMMRN2-transfected cells. 3D volume reconstruction was performed using IMARIS 9.1 software (Bitplane) in GL261 vibratome sections stained for fibronectin and CD31.

### Statistics.

Statistical analysis was performed using GraphPad Prism v6.01 software (GraphPad Software). Results are presented as mean ± SEM. Statistical differences between groups were analyzed using parametric tests including 2-tailed Student’s *t* test, 1-way ANOVA, or 2-way ANOVA, followed by Fisher’s least significant difference test for group differences. A *P* value less than or equal to 0.05 was considered significant.

### Study approval.

Human tissue was obtained in a manner compliant with the Declaration of Helsinki. The Ethics Review Board in Uppsala approved the use of human samples, and participation of patients occurred after informed consent. Use of tumor tissue microarrays and analysis of human grade IV glioma was granted by Uppsala County’s ethical committee (Dnr Ups 02-330, Ups 06-084, Dnr Ki 02-254, Ups 03-412/2003-10-02, Dnr 2010/291/2010-11-17).

All animal work was performed according to the guidelines for animal experimentation and welfare provided by Uppsala University and approved by the Uppsala County regional ethics committee (Dnr C26/15, Dnr C1/14).

## Author contributions

AD and RL designed research studies. RL, KV, and DY conducted experiments. RL, KV, AS, and MB acquired data. RL, KV, and AD analyzed data. SJ, ED, and ME provided reagents. KV, ED, SJ, and ME edited the manuscript. AD and RL wrote the manuscript.

## Supplementary Material

Supplemental data

## Version 1 05/15/2018

In-Press Preview

## Version 2 06/25/2018

Electronic publication

## Version 3 08/01/2018

Print issue publication
