# Supplementary material for: CD93 promotes β1 integrin activation and fibronectin fibrillogenesis during tumor angiogenesis
Source: J Clin Invest. 2018 Jun 25;128(8):3280–97. doi: 10.1172/JCI97459 (PMC6063507; doi:10.1172/JCI97459)
Supplement: Supplemental data [file jci-128-97459-s092.pdf]

Supplemental Data for:

CD93 promotes β_1 integrin activation and fibronectin fibrillogenesis during tumor angiogenesis

Roberta Lugano[1], Kalyani Vemuri[1], Di Yu[1], Michael Bergqvist[2,3], Anja Smits[4,5], Magnus Essand[1], Staffan Johansson[6], Elisabetta Dejana[1,7], Anna Dimberg[1]

[1] Department of Immunology, Genetics and Pathology, Science for Life Laboratory, Uppsala University, The Rudbeck Laboratory, 75185 Uppsala, Sweden

[2] Centre for Research and Development, Uppsala University, Gävle Hospital, 801 87, Gävle, Sweden

[3] Department of Radiation Sciences and Oncology, Umeå University Hospital, 901 87, Umeå, Sweden

[4] Department of Neuroscience, Neurology, Uppsala University, 75185 Uppsala Sweden

[5] Institute of Neuroscience and Physiology, Department of Clinical Neuroscience, Sahlgrenska Academy, University of Gothenburg, 41345 Gothenburg, Sweden

[6] Department of Medical Biochemistry and Microbiology, Uppsala University, Uppsala, Sweden

[7] Vascular Biology Unit, FIRC Institute of Molecular Oncology, Milan 20129, Italy

Corresponding author: Anna Dimberg, Department of Immunology, Genetics and Pathology, Rudbeck Laboratory, Uppsala University, SE-751 85 Uppsala, Sweden.

Phone: +46 702166496, Email: Anna.Dimberg@igp.uu.se

Supplemental Material and Methods:

Tumor cell and primary endothelial cell culture

GL261 glioma cells (kind gift from Dr. Geza Safrany, NRIRR, Budapest) were cultured in Dulbecco's Modified Eagle Medium (Life Technologies, Carlsbad, CA, USA) supplemented with 10% foetal bovine serum (FBS; Sigma-Aldrich, St. Louis, MO, USA) at 37°C and 5%CO_2/95% air in a humidified chamber.

Human dermal microvascular endothelial cells (HDMEC) or human dermal blood lymphatic free endothelial cells (HDBEC), purchased from PromoCell, Heidelberg, Germany were cultured up to passage 10 on gelatin-coated culture dishes in Endothelial Cell Basal Medium with full supplements (EBM-MV2, PromoCell) at 37°C and 5%CO_2/95% air in a humidified chamber.

Primary mouse brain endothelial cells were isolated from 12 weeks old C57Bl/6 wild-type or CD93$^{-/-}$ mice as previously described (49). Briefly, blood capillary fragments were seeded on collagen-I (BD Biosciences) coated wells and cultured in DMEM (Life Technologies) supplemented with 20% foetal bovine serum (FBS; Promocell) supplemented with 100µg/ml heparin (Sigma Aldrich) and 5µg/ml endothelial cell growth supplement from bovine neural tissue (ECGS, Sigma Aldrich, E2759). After 2 days of puromycin selection (4µg/ml) cells were fixed for immunolabeling and confocal microscopy analysis. Mycoplasma test was routinely performed in all cell cultures used in this study.

Co-immunoprecipitation and mass spectrometry analysis

Co-immunoprecipitation to identify CD93-interacting proteins was performed using the Pierce co-immunoprecipitation kit (Thermo Scientific) according to the manufacturer´s instructions. Briefly, total protein extract (300µg) obtained from subconfluent HDMEC were immunoprecipitated with 10µg of mouse anti human-CD93 antibody (MBL Life science, D198-3) or negative control mouse IgG (SantaCruz, sc-2025). Protein concentration was measured using the BCA Protein Assay Reagent kit (Pierce). CD93 co-immunoprecipitated samples and IgG negative controls of 5 independent experiments were analyzed by mass spectrometry for protein identification. Briefly, samples were

acetone precipitated, in-solution-digested with trypsin according to a standard protocol and analyzed by LC-Orbitrap MS/MS at the MS Facility, SciLifeLab, Uppsala University. The peptides were separated in reversed-phase on a C18-column and electrosprayed on-line to an LTQ-Orbitrap Velos Pro ETD mass spectrometer (Thermo Finnigan). Tandem mass spectrometry was performed applying CID. Protein identifications for all 10 RAW data files (5 CD93 co-IP samples and 5 IgG co-IP samples) were obtained by a database search using the quantitation software MaxQuant 1.5.1.2. The database contained human proteins extracted from UniProtKB/Swiss-Prot. A decoy search database, including common contaminants and a reverse human database, was used to estimate the identification false discovery rate (FDR). The search criteria for protein identification were set to at least two matching peptides of 95% confidence level per protein. A label free intensity analysis was performed for each individual sample. Proteins identified in at least 3 independent experiments and that were uniquely identified in CD93 co-immunoprecipitated samples and never observed in the negative control IgG were considered as potential CD93 interacting proteins.

Proximity ligation assay (PLA)

PLA was performed using the Duolink II kit (Sigma Aldrich, DUO92104) according to a standard protocol at PLA Proteomic Facility SciLifeLab, Uppsala University. Briefly, 4% paraformaldehyde (PFA) fixed cells were permeabilized with 0.1% Triton-X 100 in 3% BSA and blocked at 37℃ during 1 hour in blocking solution. Cells were incubated for 2 hours with mouse anti-human CD93 (MBL Life science, D198-3) and goat anti-human MMRN2 (SantaCruz, sc54021) primary antibodies or with rabbit anti-human CD93 (HPA, HPA009300) and mouse anti-human β_1 integrin (12G10, Abcam, ab30394) or mouse anti-human $\alpha_5\beta_1$ integrin (clone JBS5, Millipore, mab1969) primary antibodies followed by 30 minutes incubation at 37°C in ligation solution followed by 1 hour incubation with PLA secondary probes. The PLA signal was amplified using Amplification Orange and Polymerase. Cells were washed in BufferB (Olink), actin staining and nuclei detection were performed using Alexa488 Phalloidin and Hoechst respectively and mounted for microscopy analysis using SlowFade Gold Antifade Reagent (Invitrogen, S36936). Single primary antibodies with PLA probes and PLA probes without primary antibodies were routinely included as negative controls. Images were acquired

with Zeiss Axio Imager Z2 fluorescent microscope using a Plan-Apochromat 20x/0.8 M27 objective. The PLA signal was quantified in 5 different areas of the cell monolayer using the Duolink ImageTool software.

Western Blot

HDBEC were washed with PBS, lysed in NuPAGE LDS Sample buffer under reducing or non-reducing condition, according to the primary antibody manufacturer's instructions, and heated to 95°C for 5 minutes. The samples were separated onto a 4-12% Bis-Tris gel in MOPS running buffer (Life Technologies) and blotted to a nitrocellulose membrane (Immobilon). To analyze proteins secreted or cleaved from endothelial cells, conditioned medium derived from HDBEC maintained for 24 hours in EBM media supplemented with 1% FBS was collected, cell debris were removed by 3200xg centrifugation for 10 minutes. The supernatant was concentrated by precipitation with 7 volumes of acetone and total protein concentration was measured using the BCA Protein Assay Reagent kit (Pierce). 25 μg of total protein was separated onto a 4-12% Bis-Tris gel as described above. The membranes were blocked with 5% BSA or 5% skimmed milk in Tris-buffered saline (TBS) with 0.05% Tween-20 and probed with rabbit anti-human CD93 (HPA, HPA009300), mouse anti-human β_1 integrin (K20, SantaCruz sc18887), mouse anti-human CD93 (MBL Life science, D198-3), goat anti-human MMRN2 (SantaCruz, sc54021), mouse anti human β_1 integrin (12G10, Abcam, ab30394) or with a goat anti-human β–actin (SantaCruz, sc-1615), washed with 0.05% Tween-20/TBS and subsequently probed with horseradish peroxidase-labelled secondary antibodies (GE Healthcare, Chalfont St. Giles, UK and Sigma). Antibody binding was detected using the ECL Prime Western Blot detection kit (Amersham) according to the manufacturer's instructions. A representative blot image of at least 3 independent experiments is shown.

RNA extraction and qPCR

RNA from siRNA-treated endothelial cells was extracted using the RNeasy Plus Mini Kit (Qiagen). Total RNA was transcribed using Superscript III reverse transcriptase in a 20µl total volume containing 250ng of random hexamers and 40 units of RNAse OUT inhibitor (Life Technologies). mRNA expression of CD93 and MMRN2 were quantified relative to HPRT by real-time PCR in

duplicate reactions per sample with 0.25µM forward and reverse primer in SYBR Green PCR Master Mix (Life Technologies). Primer sequences are the following:

human CD93$_{forward}$ GCCCCAGAATGCGGCAGACA

human CD93$_{reverse}$ GCAGTCTGTCCCAGGTGTCGGA

human MMRN2$_{forward}$CTGCCTGGTAACCTCACAGC

human MMRN2$_{reverse}$ CTCCAAGGATCTATCAGGGAACT

human HPRT$_{forward}$ CTTTGCTGACCTGCTGGATT

human HPRT$_{reverse}$ TCCCCTGTTGACTGGTCATT

human FN$_{forward}$ GAGCAGGGACGCTCCCCAGA

human FN$_{reverse}$ TGCCATGATGACTGCCGGTCT

mouse FN$_{forward}$ CCGGTGGCTGTCAGTCAGA

mouse FN$_{reverse}$ CCGTTCCCACTGCTGATTTATC

mouse HPRT$_{forward}$CAAACTTTGCTTTCCCTGGT

mouse HPRT$_{reverse}$ TCGAGAGGTCCTTTTCACC

Embryoid bodies culture

Mouse embryonic stem (ES) cell culture and formation of embryoid bodies (EBs) was performed as described previously (52). Briefly, ES cells were cultured in Dulbecco’s minimum essential medium (DMEM)/glutamax (Gibco), 25mM Hepes (Gibco), 1.2mM sodium pyruvate (Gibco), and 19mM monothioglycerol (Sigma), supplemented with 15% FBS and 1000U/ml LIF (Chemicon). At day 0, 1200 cells were aggregated in hanging drops (20µl) without LIF. At day 4, the EBs were placed on 8-well chamber slides (2D EB culture) or on top of a polymerized collagen I gel (3D EB culture) composed of collagen I (1.5mg/ml; Inamed Biomaterials) in Ham’s F12 medium (PromoCell), 6.25mM NaOH, 12.5mM Hepes, 0.073% $NaCO_3$, and 1% Glutamax (Gibco/Invitrogen) and subsequently covered with a second layer of the collagen I mix. Four hours later, medium supplemented with VEGF-A165 (30ng/ml) was added. Medium was changed every other day and at

day 14, EBs were fixed in 4% PFA and processed for whole-mount immunofluorescence. A representative image of at least 5 individual EBs is shown. In specific experiments, ES cells were transduced with lentiviruses with CD93 or MMRN2 shRNA construct or empty vector as control. GFP-positive cells were isolated by fluorescent activated cell sorter. Transduced ES cells were cultured as described above.

Immunofluorescent staining of endothelial cells

siRNA-transfected HDBEC or mouse brain endothelial cells were plated on 8-well chamber slides, grown until confluency and then fixed in 4% PFA, either or not after a scratch was applied. After permeabilization in 1% BSA/0.1% Triton-X100/PBS and blocking in 3% BSA/0.1% Tween-20/PBS, the cells were probed with primary antibodies diluted in blocking buffer at 4 °C overnight and incubated with appropriate Alexa Fluor-conjugated secondary antibodies (Invitrogen) diluted in blocking buffer at room temperature for 2 hours. Nuclei and cytoskeleton were stained with Hoechst and Alexa Fluor 647-labelled or Texas Red-conjugated phalloidin respectively (all from Life Technologies). After that, cells were washed three times with PBS, mounted with Fluoromount-G (0100-01, Southern Biotech) and kept in 4 °C for imaging.

The following primary antibodies were used: anti-CD93 (MBL Life science, D198-3), anti-CD93 (HPA, HPA009300), anti-MMRN2 (SantaCruz, sc54021), anti-fibronectin (Abcam, ab2413), anti-fibronectin (Sigma Aldrich, F7387), anti-β_1 integrin (12G10, Abcam, ab30394), anti-$\alpha_5\beta_1$ integrin (clone JBS5, Millipore, mab1969), anti-$\alpha_v\beta_3$ integrin (Millipore, MAB1976); anti-pFAK (Y397, Abcam, ab39967), anti-α-tubulin (Abcam, ab52866) and anti-vimentin (Abcam, ab92547), anti-vWF (Dako, A0082), anti-caveolin-1 (CellSignaling, CST3238). Cells were analyzed using a Leica SP8 confocal microscope. A representative image of at least 5 individual immunofluorescent staining is shown.

Immunofluorescent staining of tumor sections, retina and embryoid bodies

Cryosections of human grade IV glioma and murine GL261 glioma sections were fixed in ice-cold acetone (Roche Diagnostics, Mannheim, Germany) followed by rehydration in PBS. Alternatively,

vibratome sections (80µm) from PFA-fixed cryoprotected brain tissues were air-dried and permeabilized in PBS containing 0.1% Triton-X100. Sections were blocked in PBS containing 3% BSA, and incubated with primary antibodies. The following primary antibodies were used: anti-CD31 (2H8; ThermoFisher Scientific, MA3105), anti-CD93 (R&D Systems, AF1696), anti-MMRN2 (MyBiosource, MBS2028221), anti-fibronectin (Abcam, ab2413) and anti-β_1 integrin (9EG7, BD Pharmigen, #553715). Sections were washed in PBS and stained with Alexa Fluor-conjugated secondary antibodies (Invitrogen). Nuclei were stained with Hoechst (Sigma-Aldrich), and the sections were mounted using Fluoromount-G (Southern Biotech) and kept in 4 °C for imaging. A representative image of at least 10 individual immunofluorescent staining is shown.

To analyze and quantify the retina vasculature, CD93$^{-/-}$ and wild-type as well as heterozygous littermate eyes (P6) were fixed in 4% PFA for 2 hours at 4 °C and dissected. After blocking and permeabilization in 1% BSA, 0.5% Triton-X100 in PBS overnight, retinas were incubated with anti-CD31 (2H8; ThermoFisher Scientific, MA3105) or 488-conjugated isolectin B4 (ThermoScientific, I21411), anti-ERG (Abcam,ab92513), anti-CD93 (R&D Systems, AF1696) and anti-MMRN2 (MyBiosource, MBS2028221), anti-fibronectin (Abcam, ab2413) and anti-β_1 integrin (9EG7, BD Pharmigen, #553715) primary antibodies. Then the retinas were incubated with Alexa Fluor-conjugated secondary antibodies (Invitrogen) and mounted with Fluoromount-G (Southern Biotech). A representative image of at least 10 individual retina immunofluorescent staining is shown. Quantifications were performed using ImageJ.

For whole-mount EBs immunostaining, EBs were fixed for 45 minutes in 4% PFA in PBS. After washing, samples were blocked and permeabilized for 2 hours in 3% BSA and 0.2% Triton X-100 in PBS, followed by sequential overnight incubation of primary antibodies (anti-CD31, anti-CD93 and anti-MMRN2) and 2 hours incubation with fluorescently labelled secondary antibodies. After washing, samples were mounted with Fluoromount-G (0100-01, Southern Biotech) and kept in 4 °C for imaging. Samples were analyzed under confocal microscope (Leica SP8) or fluorescent microscope Leica DMi8.

Supplemental Figures:

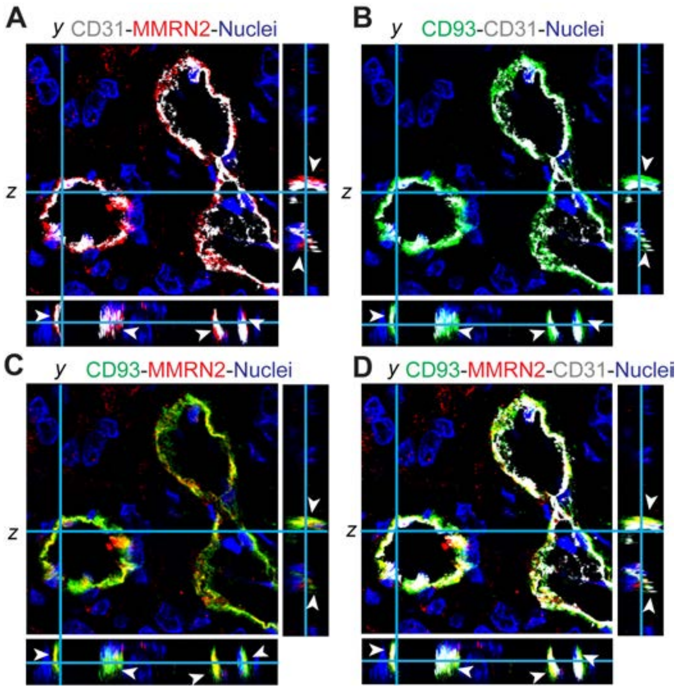

Supplemental Figure 1. CD93 and MMRN2 colocalize at the abluminal side of human grade IV glioma. y-z 3D stack of MMRN2 and CD93 co-staining in human grade IV glioma sample. Arrowheads indicate an abluminal localization of MMRN2 (**A**) and CD93 (**B**) with respect to CD31 positive vessels. (**C-D**) show co-localization between CD93 and MMRN2 in the glioma vessels. The same tissue section is shown in **Figure 1D**.

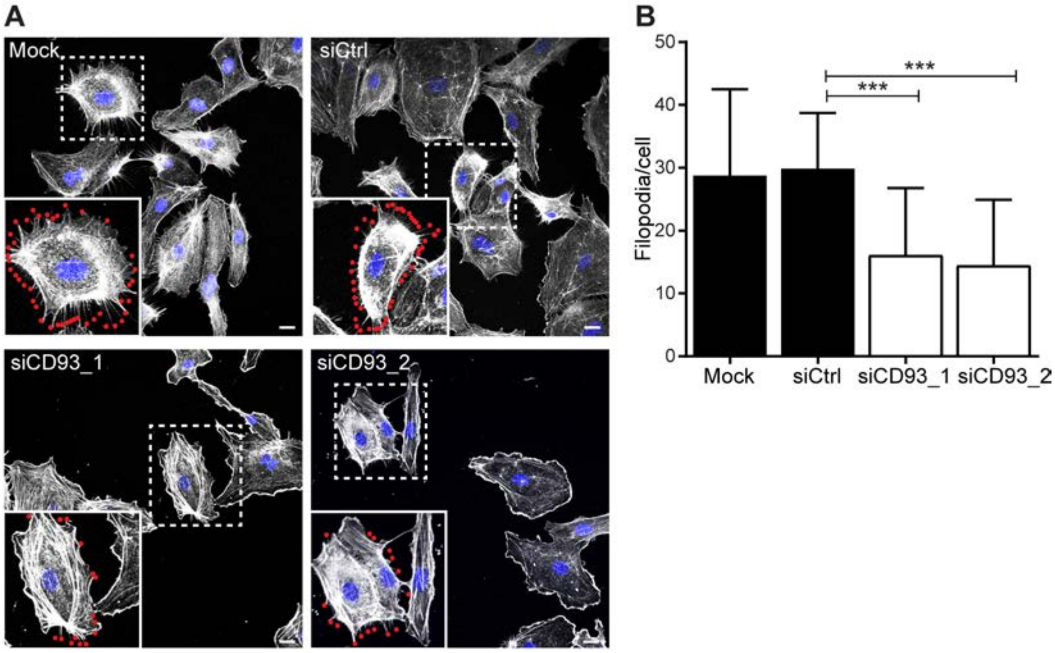

Supplemental Figure 2. CD93 knockdown affects filopodia formation in HDBEC. (A) Filopodia formation in non-confluent control (Mock; siCtrl) and siCD93 tranfected HDBEC (siCD93_1; siCD93_2). Cells were stained by phalloidin (F-actin; white). Red dots indicate filopodia protrusions. Scale bar 20µm. **(B)** Quantification of filopodia in control and silenced CD93 cells. Bars represent the number of filopodia per cell relative to siCtrl control. ***p<0.001; one-way ANOVA with Dunnett's post-test. Values represent mean±SEM.

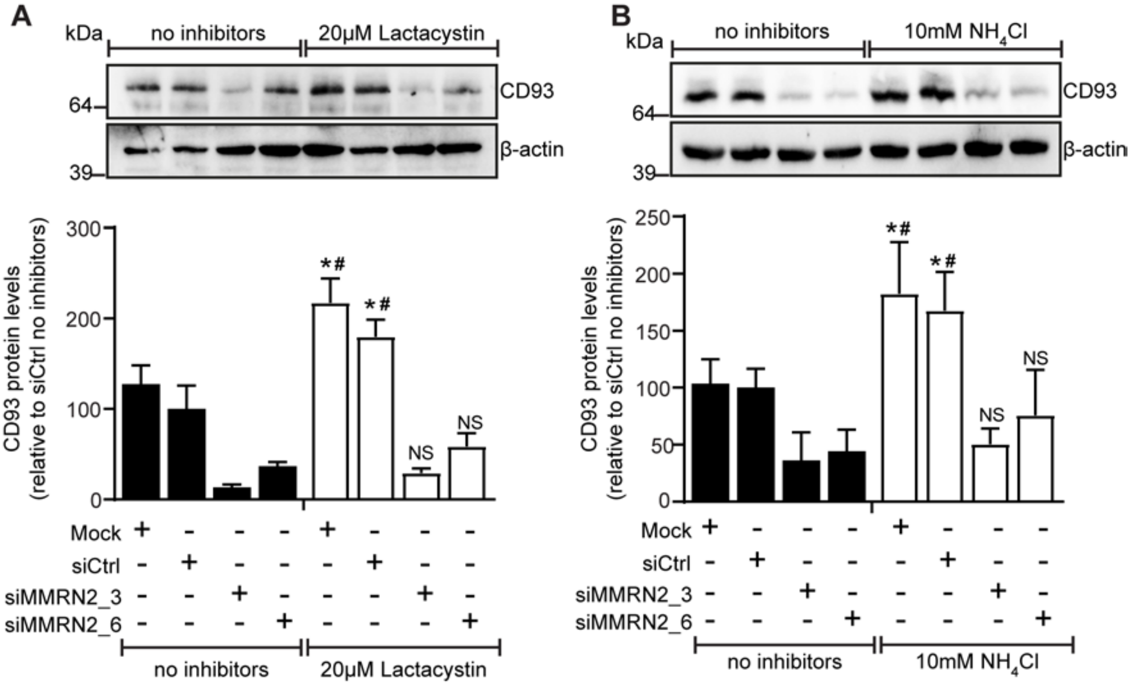

Supplemental Figure 3. MMRN2 knockdown does not affect CD93 degradation in HDBEC. (A) Western blot analysis of CD93 levels in control and siMMRN2 transfected cells after the inhibition of proteasome-mediated degradation (at 8 hours after 20µM lactacystin inhibitor treatment). Increased levels of CD93 in control cells indicate efficient inhibition of proteasome-mediated degradation. Values represent mean±SEM (3 independent experiments) *p<0.05 vs Mock; #p<0.05 *vs* siCtrl; NS= not significant *vs* the respective siMMRN2 cells; one-way ANOVA with Dunnett's post-test **(B)** Western blot analysis of CD93 levels in control and siMMRN2 transfected cells after inhibition of lysosome-mediated degradation (18 hours after 10mM NH_4Cl inhibitor treatment). Increased levels of CD93 in control cells indicate efficient inhibition of lysosome-mediated degradation. Values represent mean±SEM (3 independent experiments) *p<0.05 vs Mock; #p<0.05 *vs* siCtrl; NS= not significant vs the respective siMMRN2 cells; one-way ANOVA with Dunnett's post-test.

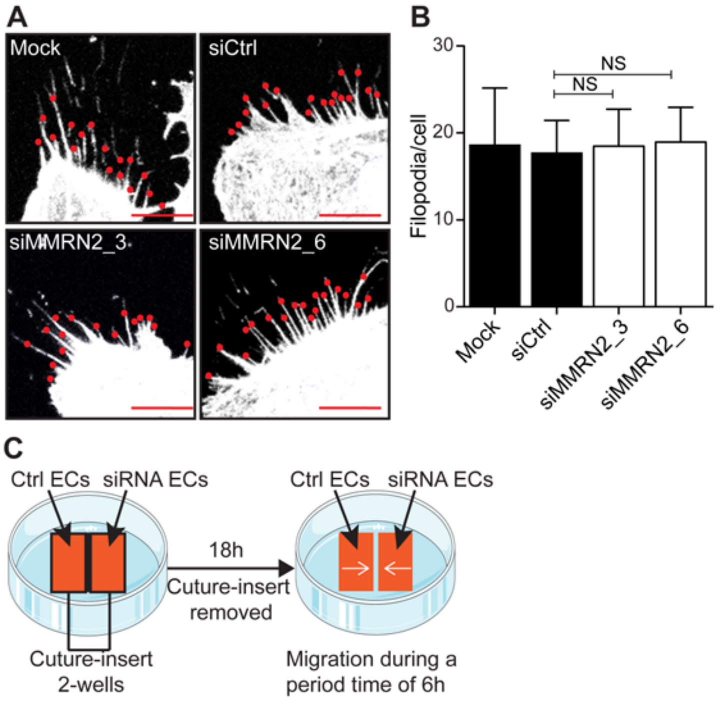

Supplemental Figure 4. MMRN2 knockdown does not affect filopodia formation in endothelial cells. (A) Filopodia formation in non-confluent control (Mock; siCtrl) and siMMRN2 transfected HDBEC (siMMRN2_3; siMMRN2_6). Cells were stained by phalloidin (F-actin; white). Red dots indicate filopodia protrusions. Scale bar 10µm. **(B)** Quantification of filopodia in control and MMRN2-silenced cells. Bars represent the number of filopodia per cell relative to siCtrl control. Values represent mean±SEM; NS=not significant; one-way ANOVA with Dunnett's post-test **(c)** Schematic representation of the culture-insert used in the CD93 rescue experiment. Control cells and siMMRN2 transfected cells were seeded in separate wells until confluence (18 hours). Culture-insert was removed. Control cells and siMMRN2 transfected cells were induced to migration with 10%FBS supplemented medium. After 6 hours of migration, cells were fixed and the CD93 levels at the migration front were assessed by immunofluorescent staining and confocal microscopy analysis.

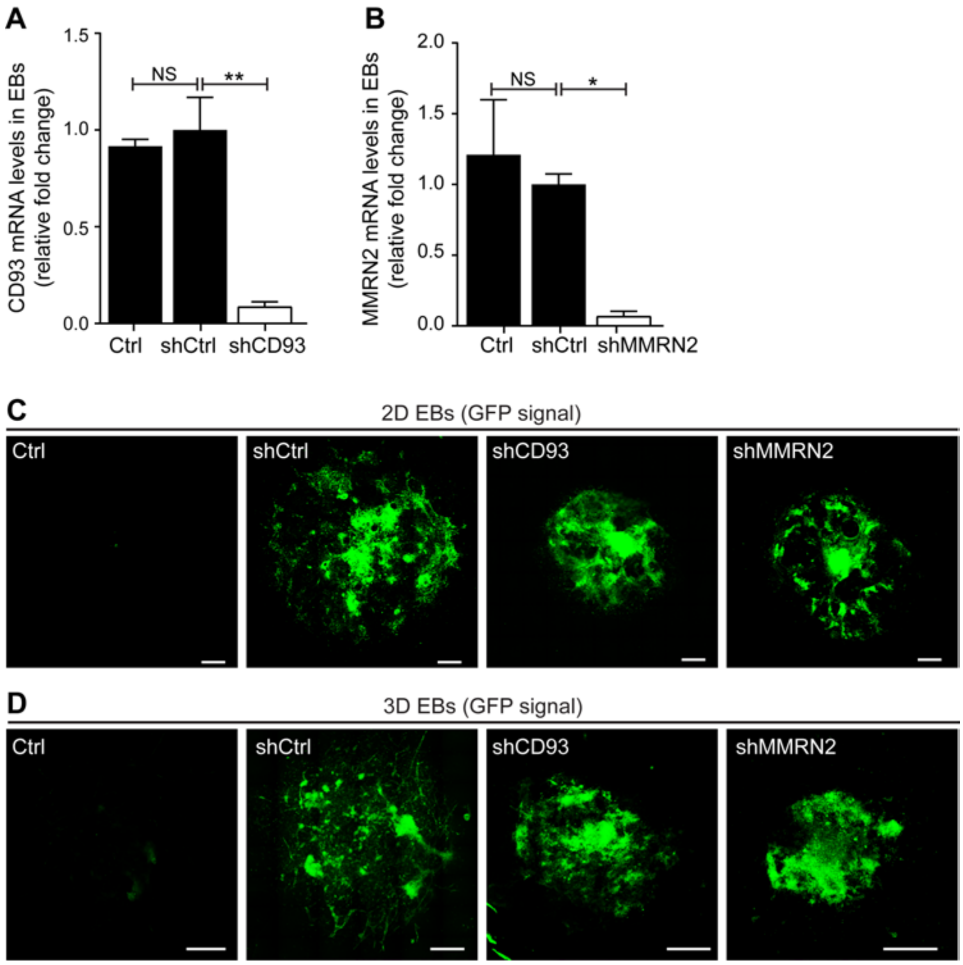

Supplemental Figure 5. CD93 and MMRN2 shRNA efficiency in EBs. (A) q-PCR analysis of CD93 mRNA expression in EBs after lentivirus transduction of shRNA sequence targeting CD93 (shCD93). (**B**) MMRN2 mRNA expression in EBs after lentivirus transduction of shRNA sequence targeting MMRN2 (shMMRN2). Untransduced EBs (Ctrl) and empty vector transduced EBs (shCtrl) were used as control. Values represent fold change relative to shCtrl transfected EBs. *$p<0.05$, **$p<0.001$; NS=not significant; one-way ANOVA with Dunnett's multiple comparison test. GFP expression in 2D-EBs (**C**) and 3D-EBs (**D**) after lentivirus transduction. Scale bar 500µm.

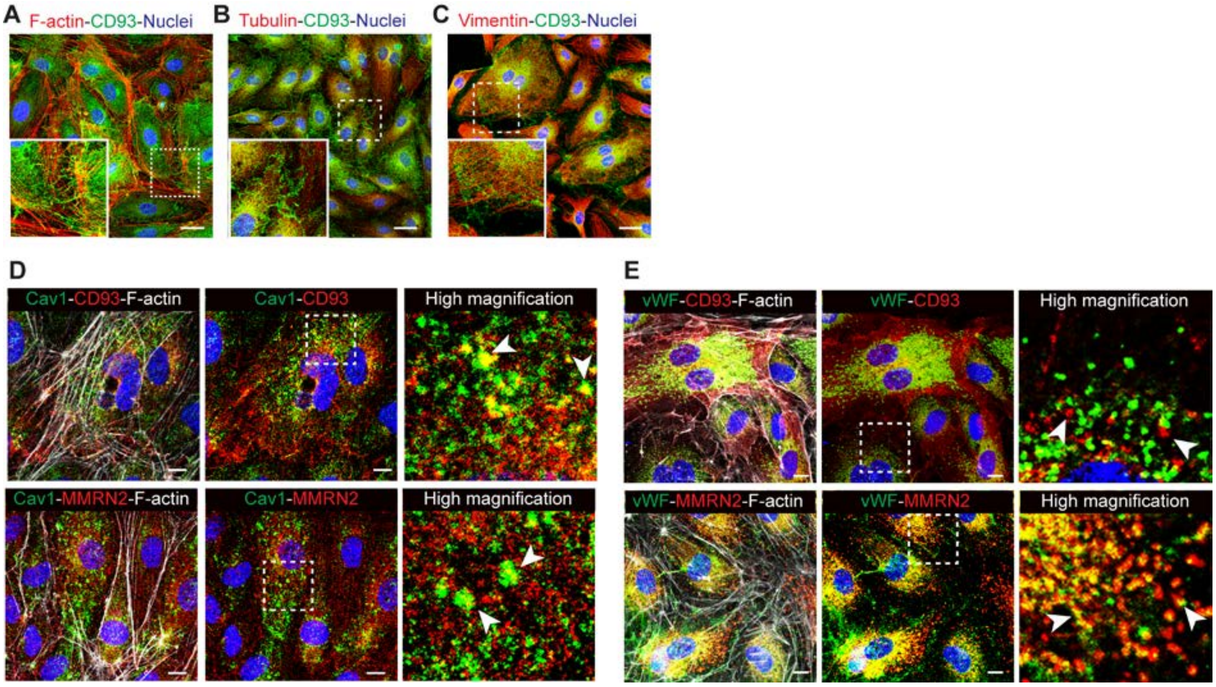

Supplemental Figure 6. CD93 and MMRN2 subcellular localization. Immunofluorescent costaining for CD93 (green) and cytoskeletal proteins (red) in HDBEC; F-actin **(A),** tubulin **(B)** and vimentin **(C)**. Scale bar: 20µm. **(D)** Immunofluorescent staining for CD93 (red, upper panels) or MMRN2 (red, lower panels) and caveolin-1 (Cav-1, green) in confluent endothelial cells. Arrowheads in high magnification pictures shown colocalization between CD93 and caveolin-1 in the CD93-Cav-1 co-staining panel (upper) and a different distribution of MMRN2 and caveolin-1 in the MMRN2-Cav-1 costaining panel (lower). **(E)** Immunofluorescent staining for CD93 (red, upper panels) or MMRN2 (red, lower panels) and vonWillebrand factor (vWF, green) in confluent endothelial cells. Arrowheads in high magnification pictures show a different distribution of CD93 and vWF in the CD93-vWF co-staining panel (upper) and strong co-localization of MMRN2 with vWF in the MMRN2-vWF costaining panel (lower). Scale bar: 10µm.

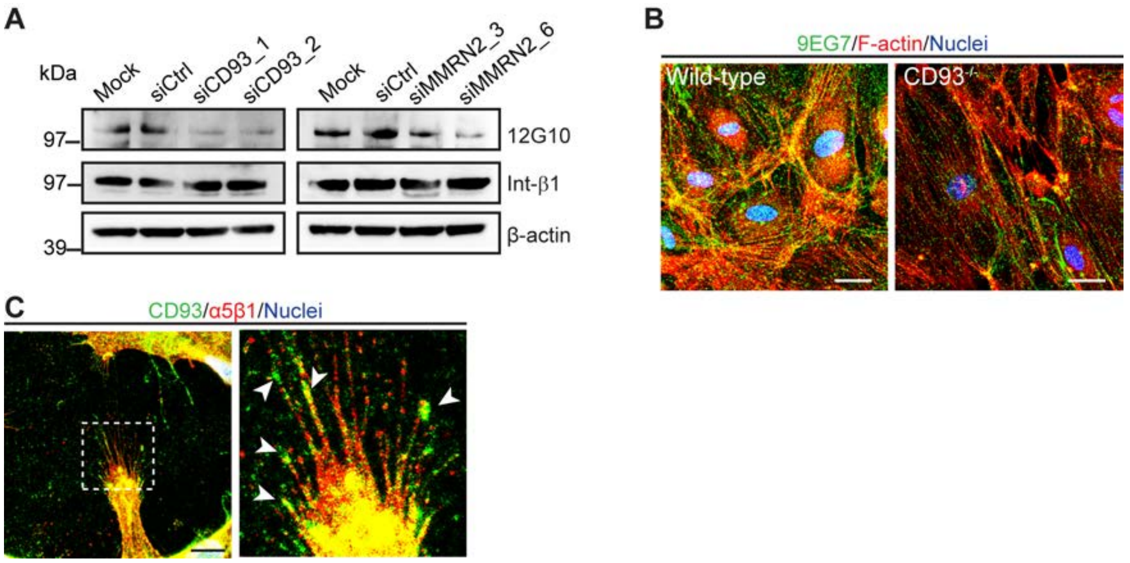

Supplemental Figure 7. CD93 and MMRN2 regulate β_1 integrin activity in endothelial cells. (A) Western blot analysis for active β_1 integrin (12G10), β_1 integrin (K20) and β-actin in control (Mock and siCtrl), siCD93-treated and siMMRN2-treated cells (siCD93_1, siCD93_2 and siMMRN2_3, siMMRN2_6 respectively). (**B**) Immunofluorescent staining of active β_1-integrin (9EG7) in brain endothelial cells derived from wild-type and CD93$^{-/-}$ mice. Scale bar: 20µm. (**C**) Immunofluorescent staining of CD93 and $\alpha_5\beta_1$ integrin in the filopodia of sparsely seeded HDBEC. Scale bar: 20µm.

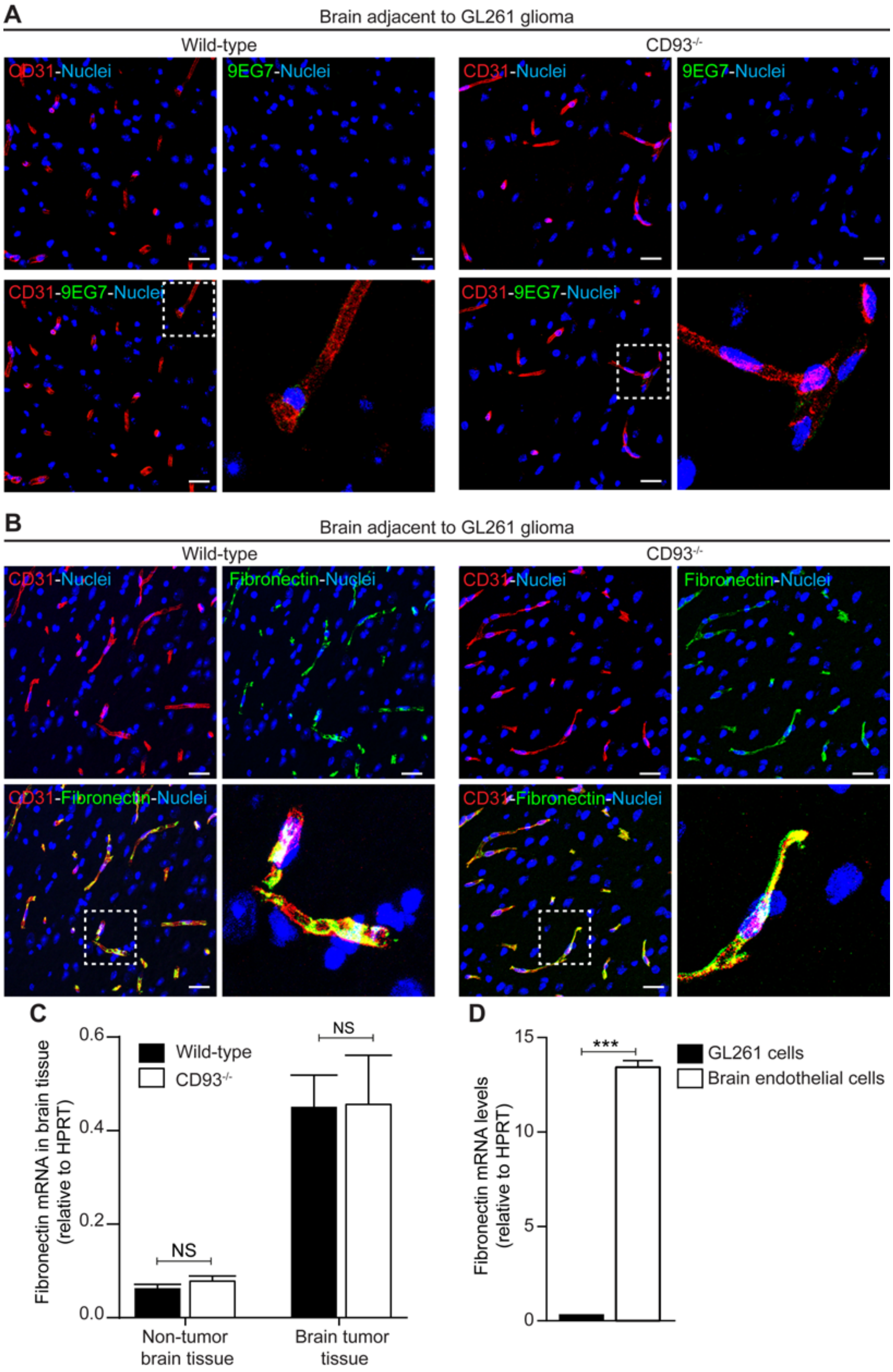

Supplementary Figure 8. Expression of active β_1-integrin and fibronectin in brain vasculature adjacent to GL261 tumor. (A) Immunofluorescent staining of active β_1-integrin (9EG7, green) and CD31 (red) in non-tumor brain vasculature of GL261 tumors intracranially implanted in wild-type and CD93$^{-/-}$ mice. Scale bar: 20µm. **(B)**. Immunofluorescent staining of fibronectin (green) and CD31 (red) in non-tumor brain vasculature of GL261-bearing wild-type and CD93$^{-/-}$ mice. Scale bar 20µm. (**C**) Real-time qPCR of fibronectin in healthy brain tissue (non-tumor) and brain tumor tissue of wild-type

and CD93$^{-/-}$ mice. Graph show upregulation of fibronectin in tumor tissue compared with non-tumor tissue and no differences in the fibronectin expression between wild-type and CD93$^{-/-}$ mice. Values represent mean±SEM (n=3 mice/group), NS= not significant. (**D**) Real-time qPCR of fibronectin in cultured GL261 cells and brain endothelial cells derived from wild-type mice. Values represent mean±SEM (n=3 independent experiments). ***p<0.0001; 2-tailed *t*-test.
